# Supplementary material for: Distinct hippocampal-prefrontal neural assemblies coordinate memory encoding, maintenance, and recall
Source: Curr Biol. 2023 Apr 10;33(7):1220–1236.e4. doi: 10.1016/j.cub.2023.02.029 (PMC10728550; doi:10.1016/j.cub.2023.02.029)
Supplement: Document S1. Figures S1–S7 [file mmc1.pdf]

Current Biology, Volume *33*

Supplemental Information

Distinct hippocampal-prefrontal neural assemblies coordinate memory encoding, maintenance, and recall

Aleksander P.F. Domanski, Michal T. Kucewicz, Eleonora Russo, Mark D. Tricklebank, Emma S.J. Robinson, Daniel Durstewitz, and Matt W. Jones

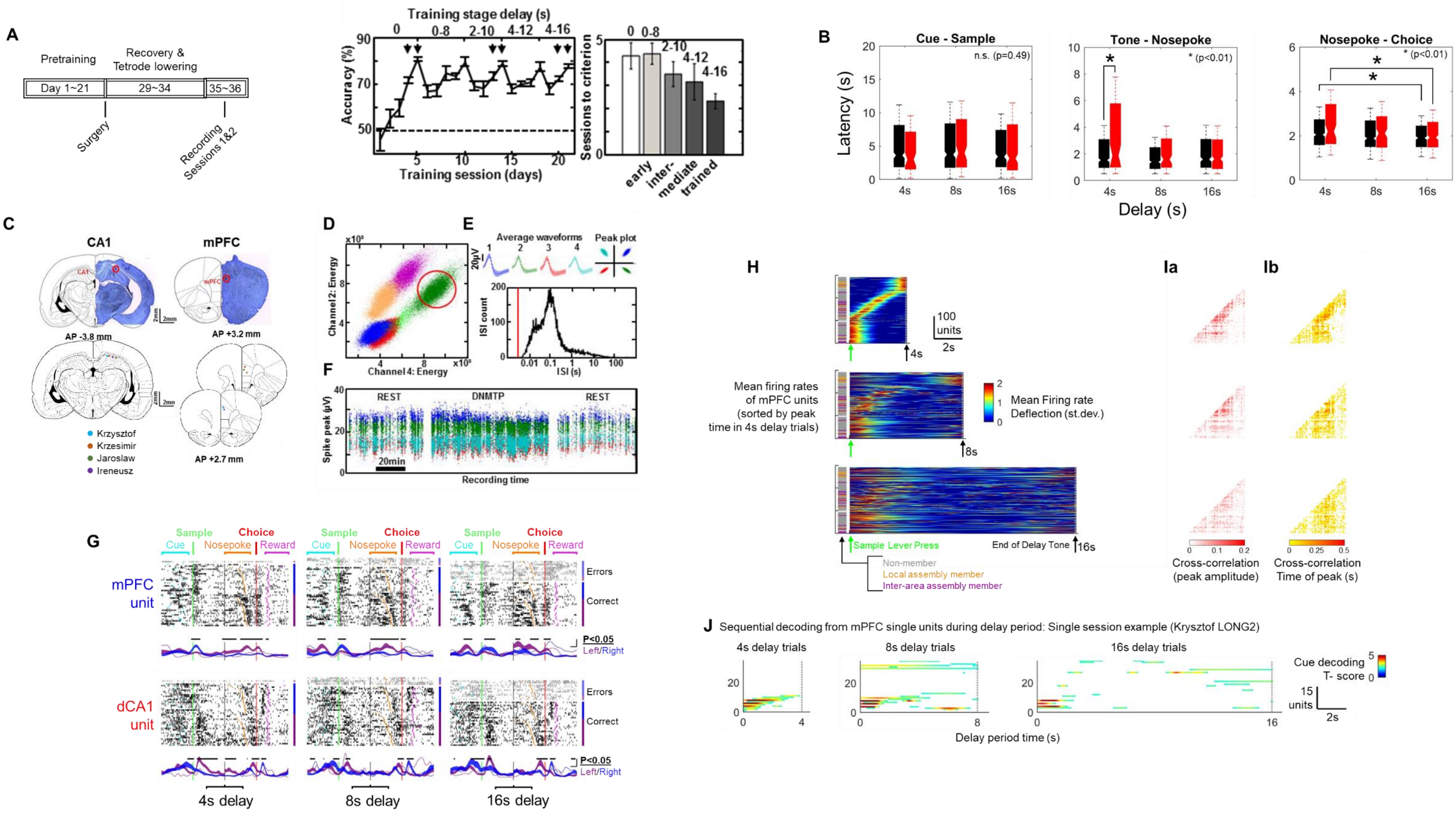

A
Pretraining
Recovery & Tetrode lowering
Day 1~21
29~34
35~36
Surgery
Recording Sessions 1&2
Training stage delay (s)
0
0-8
2-10
4-12
4-16
Accuracy (%)
Training session (days)
Sessions to criterion
early
inter-mediate
trained
B
Cue - Sample
n.s. (p=0.49)
Tone - Nosepoke
* (p<0.01)
Nosepoke - Choice
* (p<0.01)
Latency (s)
Delay (s)
4s
8s
16s
C
CA1
mPFC
AP -3.8 mm
AP +3.2 mm
AP +2.7 mm
2mm
Krzysztof
Krzesimir
Jaroslaw
Ireneusz
D
Channel 2: Energy
Channel 4: Energy
E
Average waveforms
Peak plot
20µV
ISI count
ISI (s)
F
Spike peak (µV)
REST
DNMTP
REST
20min
Recording time
G
mPFC unit
dCA1 unit
Sample
Choice
Cue
Nosepoke
Reward
Errors
Correct
P<0.05
Left/Right
4s delay
8s delay
16s delay
H
Mean firing rates of mPFC units (sorted by peak time in 4s delay trials)
100 units
2s
Mean Firing rate Deflection (st.dev.)
Sample Lever Press
End of Delay Tone
Non-member
Local assembly member
Inter-area assembly member
Ia
Ib
Cross-correlation (peak amplitude)
Cross-correlation Time of peak (s)
J Sequential decoding from mPFC single units during delay period: Single session example (Krysztof LONG2)
4s delay trials
8s delay trials
16s delay trials
Cue decoding T- score
15 units
Delay period time (s)

Figure S1: Details of training, recordings and single unit physiology during DNMTS task (relating to Figure 1).

A Left: Experimental timeline, Center: Behavioral performance of rats in DNMTS training is expressed as choice accuracy across subsequent sessions of the training and as average number of sessions required to reach criterion performance at different stages of training: early (Right). Arrows mark the two sessions used in the analysis of each stage (early, intermediate, trained). Data from the "trained" days is analysed in this study.
B Cue-Sample, Delay-Nosepoke, Nosepoke-Choice latencies averaged across animals and recording sessions for correct (black) and error (red) trials. Asterisks at upper right corner indicate significant difference between conditions (Kruskal-Wallis ANOVA: χ!(5,1751)=3.69/16.29/16.53, $p>0.05$/$p<0.01$/$p<0.01$, respectively). Bars/asterisks indicate significant differences between specific combinations of delay and outcome conditions (Tukey-Kramer post-hoc test, $p<0.05$).
C Coronal sections show example locations of tetrode recording sites (red circles mark the site of electrolytic lesions) in the prelimbic cortex (mPFC) and in the pyramidal cell layer of the dCA1 subfield in dorsal hippocampus (dCA1), matched to a corresponding rat brain atlas schematic (from Paxinos 2008). The lower panels summarize lesion sites across all six rats.
D Extracellular action potential spikes recorded across an entire session were clustered into separate single units (colored dots), plotted here as waveform energy recorded on two channels of one mPFC tetrode. The properties of the cluster in red circle are presented in E and F.
E Mean waveforms recorded on color-coded channels of the tetrode (top left) show consistent relative peak amplitudes (top right). Distribution of inter-spike intervals (ISI) below show no spikes detected in the <2ms refractory period.
F Spike peak amplitudes of the same unit recorded on the color-coded four tetrode channels remain stable across the recording session.
G Multi-trial firing raster from one example mPFC (top) and dCA1 (bottom) single units. Spike rasters with continuous firing rates aligned to the Sample and Choice lever presses +/-5s, with a variable portion of the delay period excised depending on delay length. Trials are sorted by correct and error outcomes (black and grey ticks) as well as left and right trial type, and finally by nose-poke latency. Solid areas indicate mean +/-SEM firing rates on correct trials, dotted lines show mean firing rate on error trials. Black bars above epochs show significant separation of Left/Right trial responses, from the trial-averaged firing rates (t-score, in 50ms non-overlapping increments, Bonferroni-corrected).
H Mean firing rates of mPFC units (preferred cue direction, correct trials, all units across sessions combined) during the delay period sorted by time of peak firing on 4s delay trials, sort order maintained for 8,16s delay trials. Colored stripes on Left indicate assembly membership class (See Figures 3,4).
I Firing rate correlation matrices (Ia: peak correlation and Ib: time-lag at peak) for data shown in H. Heat-map shows mean correlation across trials, note strong non-zero lagged correlations.
J Sequential contributions of individual mPFC single units to maintaining population-level encoding of cue location during maintenance delay. Single recording session shown. Units are shown sorted by center-of-mass of significant decoding (bootstrapped Bonferroni $p<0.05$) on 4s delay trials. Sort times maintained across longer delay lengths. Colourmap as for Figure 1D.

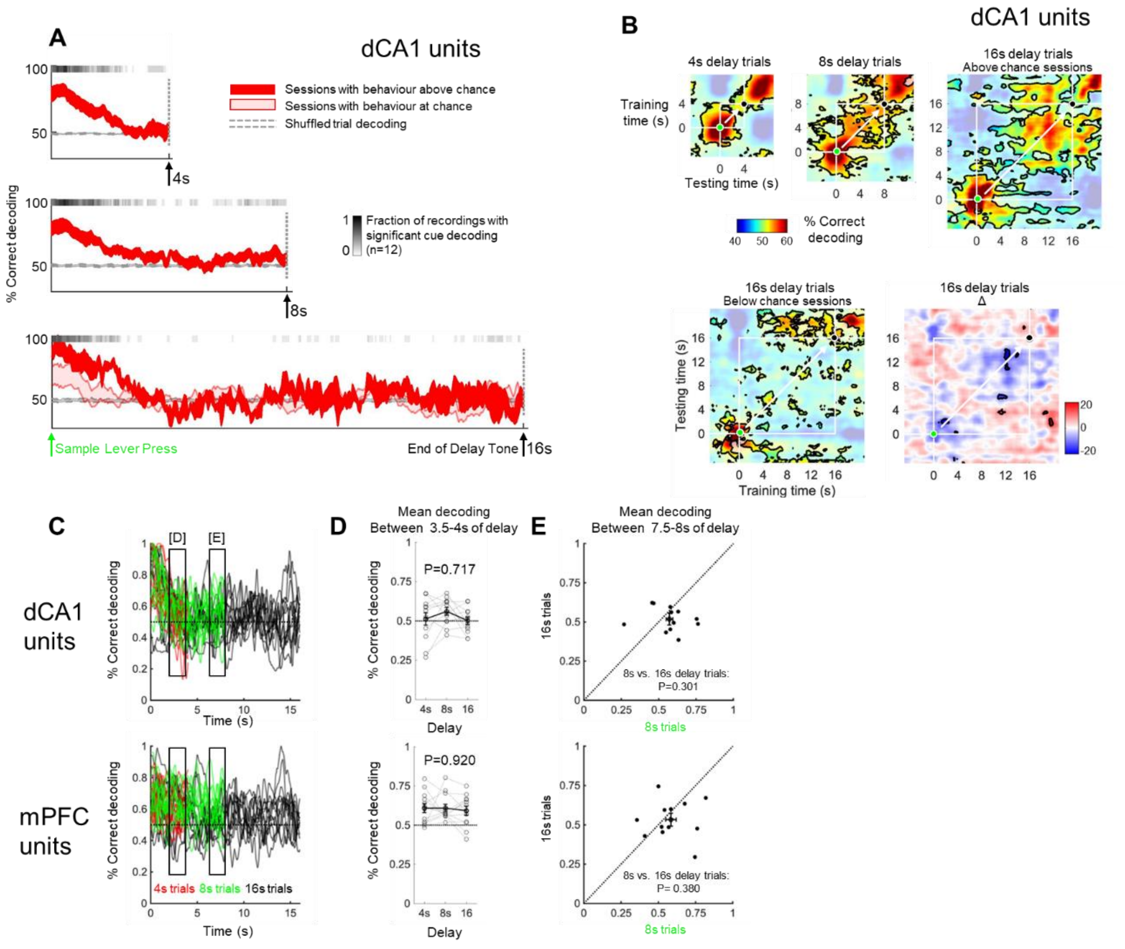

Figure S2: Transient cue encoding in dCA1 population lacks a stable code, further details of delay coding in dCA1 and mPFC (relating to Figure 2).

A-B: Encoding of cue information during the delay. Legends as for Figure 2 but decoding from populations of dCA1 single units.

C: Delay-dependent performance of dCA1 and mPFC populations for each session, regardless of whether the rat was performing above or at chance for that delay length (each line represents on session). Black boxes indicate the 0.5s windows used to calculate the average decoding performance per dCA1/mPFC population around 4s and 8s during the delay (shown in D and E, respectively).

D: No trial-dependent differences in average decoding between 3.5-4s during the delay were detected in either brain area. Linked symbols are recording sessions, linked by delay length. Error bars indicate mean±SEM (Friedman's test: dCA1: C_2(2,22)=0.67, p=0.717; mPFC: C_2(2,22)=0.17, p=0.920).

E: No trial-dependent differences in average decoding between 7.5-8s during the delay were detected in either brain area. Linked symbols are recording sessions, linked by delay length. Error bars indicate mean±SEM. Paired Wilcoxon dCA1: T= 25, z = -1.10, p=0.301, mPFC: T = 32, z = -0.550,P=0.380.

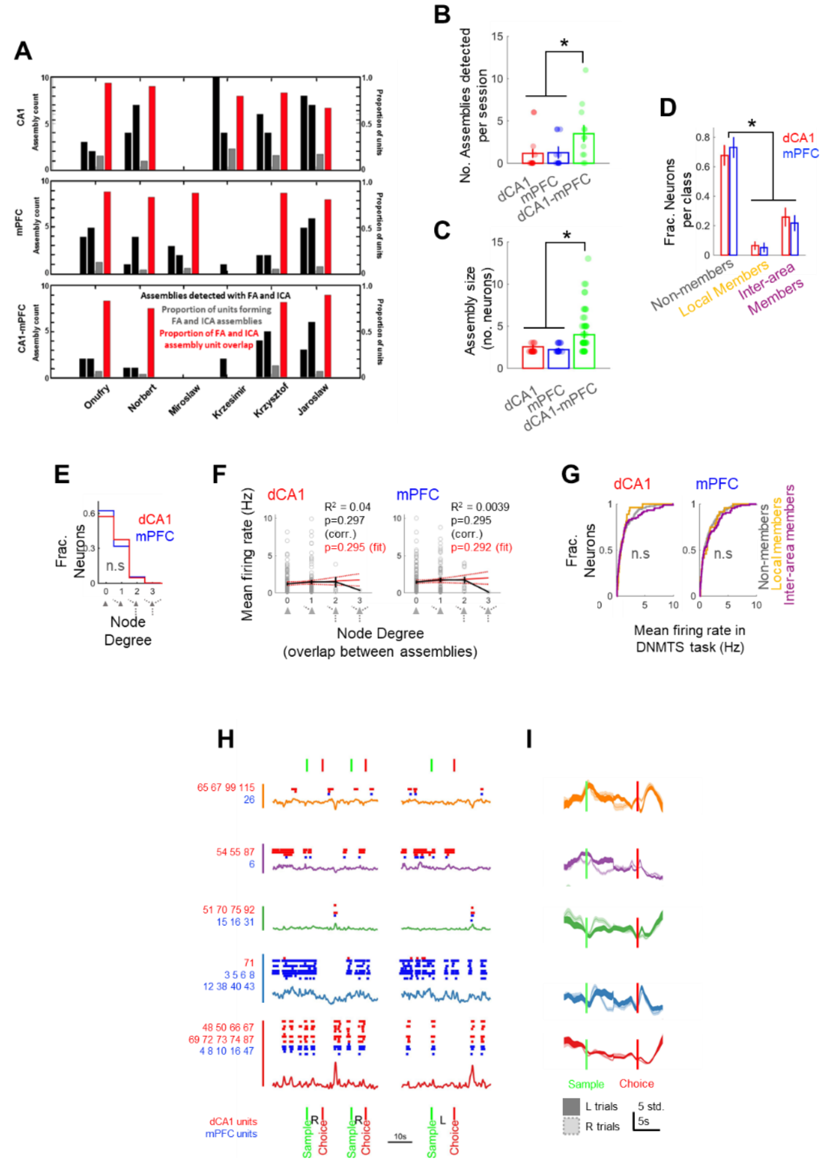

Figure S3: Further details of detected local and inter-regional dCA1-mPFC cell assemblies (relating to Figure 3).

A Validation of assemblies detected with the FA against PCA-ICA based methods. Total number of assemblies detected (black bars – the first for FA, second for ICA), proportion of units that participated in assemblies detected by both FA and ICA relative to all units recorded (grey), and proportion of unit overlap between matched FA and ICA assembly pairs (red) are summarized for the two sessions of each rat. A measure of overlap between a pair of assembly sets A and B was formally defined as O=|AÇB|/|AČB|î [0,1], i.e. the cardinality of the intersection divided by the cardinality of the union. On average there was 84% overlap between units detected by the FA and ICA methods, with similar numbers for total counts and unit proportions involved.

B Detection rates for local and inter-regional classes of cell assemblies. Symbols are individual sessions (1.2±0.5/1.3±0.7 dCA1/mPFC vs. 3.5±0.9 joint dCA1-mPFC Kruskal-Wallis test: C2(2,33)=6.69 p=0.035 with Bonferroni-correct post-hoc test).

C Size of detected cell assemblies, for dCA1/mPFC/inter-area classes, ANOVA F(2,16)=6,2,N=71 p=0.01).

D Breakdown of units by assembly membership class A significant minority of units participated in cell assemblies (ANOVA for member vs, non-member, F(5,66)=32.3, p<0.01, local membership: 6±3%/5±3%; dCA1/mPFC vs inter-area membership 26±3%/22±5% of dCA1/mPFC neurons). Assembly membership was similarly sparse in both dCA1 and mPFC area: Fraction of members vs. non-members dCA1 vs mPFC: C2(1)=0.33, p=0.94.

E The majority of neurons forming cell assemblies did so with little overlap between membership: 73%/67% of dCA1/mPFC units were detected as members of only a single assembly, with no differences in membership orthogonality observed between the two areas

(Figure 3F, KS test for node degree of mPFC vs. dCA1 neurons, D=0.045, p=0.87, N=295;317, dC1;mPFC).

F Mean firing rates of units calculated across the duration of the task were not affected by membership degree (Figure 3G, red curves: Linear fit vs. no relationship, dCA1: F(270)=1.09, p=0.30, adjusted R_2=3x10_{-4}; F(287)=1.11, p=0.29, adjusted R_2=4x10_{-4}). Not shown: Firign rate did not depend on global assembly membership (Mann-Whitney U-test for mean firing rate of members vs non-members: p=0.6,p=0.63 for dCA1,mPFC neurons, respectively, N=295,317 neurons).

G Classifying neurons as non-members, local members or inter-area cell assembly members did not significantly partition mean firing rate distributions (Figure 3H, Kruskal Wallis test: dCA1: c_2(2,292)=0.29, p=087; mPFC: c_2(2,314)=0.33, p=0.86). Similar results were obtained from surrogate measure of optimal firing rate smoothing kernel (data not shown).

H Activities of co-occurring inter dCA1-mPFC cell assemblies from example recording shown in Figure 3C-D. Legend as for Figure 3D: units from each area are shown concatenated.

I Normalized trial-averaged activity of assemblies in H during sample and choice lever events, for Left and Right cue trials. Mean±SEM activity across trials shown.

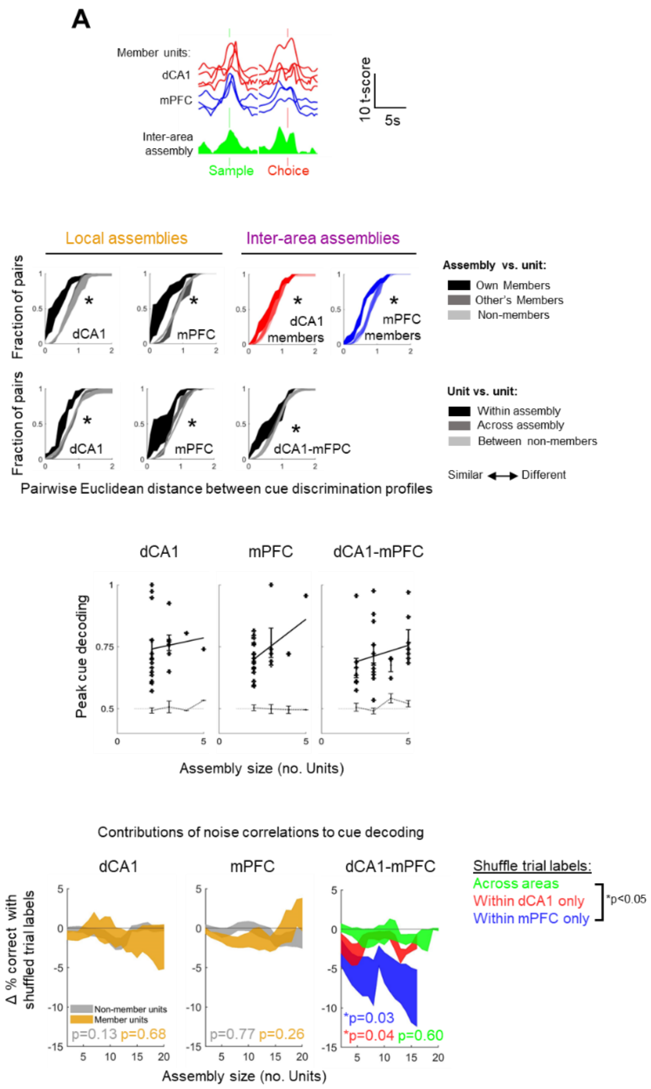

Figure S4: Cell assemblies link units carrying similar information (relating to Figure 4).

A Cue location decoding profiles of an example dCA1-mPFC assembly and its constituent member units aligned to Sample and Choice lever presses.
B Distributions of pairwise Euclidean distances between cue-decoding profiles of pairs of units/assemblies, sorted by assembly membership comparison type. In all cases, distances are closer (temporal evolution is more similar) for within- than between-assembly comparisons (Mean±SEM across sessions shown, asterisks indicates Kruskal-Wallis test, $p<0.05$).
C Expanded x range of Figure 4F, showing relationship between cell assembly size and peak cue-decoding.
D Contributions of within-trial (‘noise’) firing rate correlations to performance of optimally aggregated cell assemblies shown in Figure 4F. Mean±SEM peak performance change of best performing synthetic cell assemblies after shuffling trial labels, keeping cue location labels intact (Negative: reduced decoding performance after removing within-trial correlations). Colored p-values indicate one-sample t-test results: Within-area: dCA1 non-members: $t(7)=-1.21$, $p=0.13$; members: $t(8)=0.42$, $p=0.68$; mPFC non-members: $t(11)=-0.29$, $p=0.77$; members: $t(11)=-1.63$, $p=0.26$. Inter-area: within-dCA1: $t(7) =-2.75$, $p=0.04$; within-mPFC: $t(7) = -2.63$, $p = 0.04$; cross-dCA1-mPFC: $t(9) = -0.54$, $p=0.60$. Right: contributions of within-trial firing rate correlations of inter-area assembly performance were significantly different within and across areas (ANOVA: $F(2,23)=4.02$, $p=0.032$); cue decoding after ablation within mPFC pool caused significantly greater impairment than across dCA1-mPFC correlations (Asterisk:Tukey-Kramer post-hoc test, $p<0.05$).

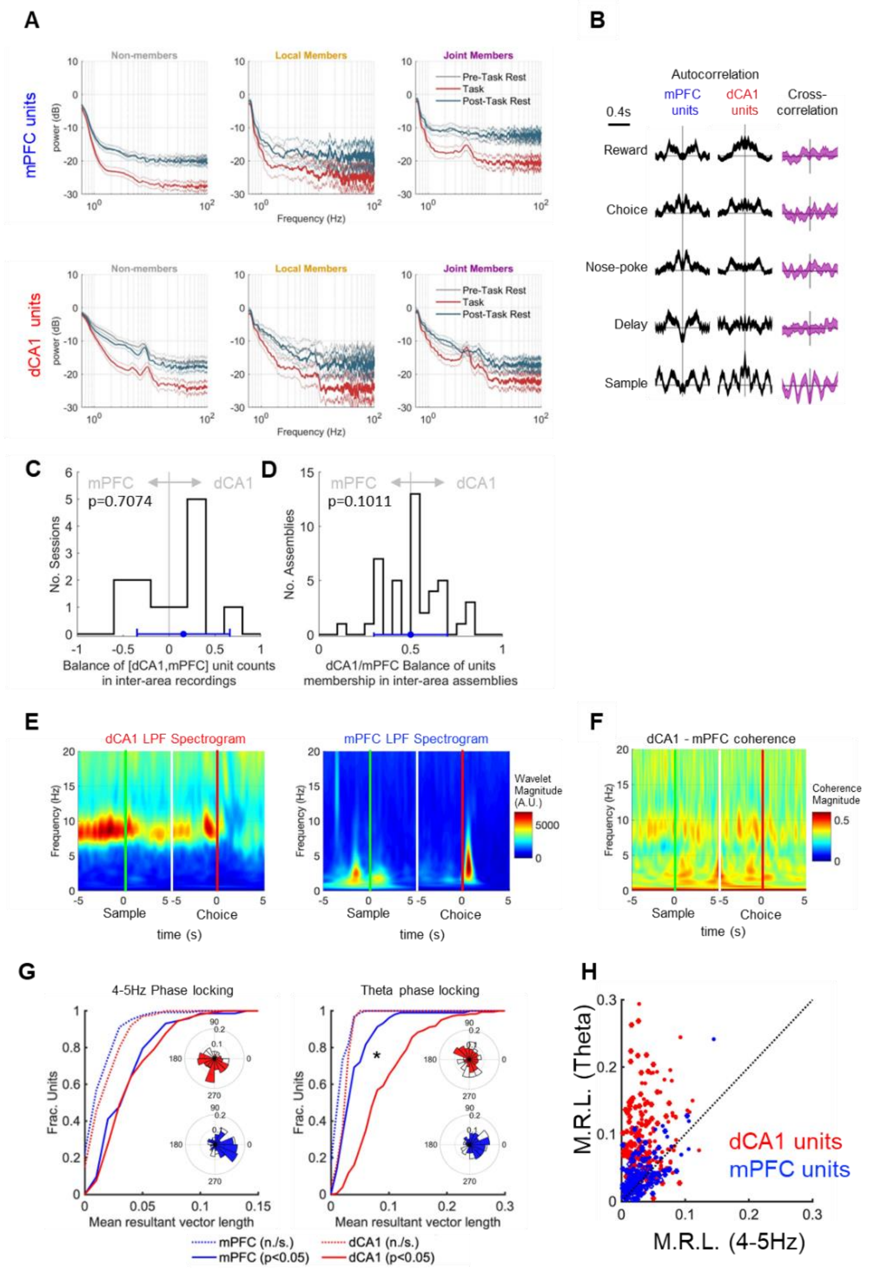

Figure S5: Physiological details of dCA1-mPFC cell assemblies (relating to Figure 5).

A Power spectral densities of spike time autocorrelations for units of each area sorted by assembly membership category. Curves show mean±SEM spectra, Spikes are drawn from either the task period (red), or 1h pre- and post-task rest periods (grey, teal). For both dCA1 and mPFC units, the 4-5Hz oscillation was specific to the task period and, in mPFC, to crossregional assembly members.

B Left: Z-scored spike time autocorrelation (black) and cross-correlation (purple) functions of dCA1 and mPFC units restricted to task events. Lines show mean±SEM.

C Units from both regions are equally represented in our recordings: Histogram across sessions of ratios between single unit counts in multi-area recordings $[(\#units_{"\#\$\%} - \#units_{\&\%'()})/(\#units_{"\#\$\%} + \#units_{\&\%'()})]$. Ratio distribution was not significantly different from a normal distribution (Kolmogorov-Smirnoff test). Blue symbols indicate median ± inter-quartile range.

D Inter-area cell assemblies are equally contributed by dCA1 and mPFC single units. Ratio distribution was not significantly different from a normal distribution with mean=0.5. (Kolmogorov-Smirnoff test). Blue symbols indicate median ± inter-quartile range.

E Mean wavelet spectrograms of dCA1 (left) and mPFC (right) LFP, aligned to the sample and choice lever press events in the task. Average of one tetrode per session for each area.

F As for E, but showing wavelet coherogram between dCA1 an mPFC LFP signals.

G Single unit phase locking to 4-5Hz (left) and 8-12Hz (“theta”, right) LFP oscillations during

the DNMTS task. Cumulative histograms show distributions of phase locking strengths (mean resultant length of phase vectors) for units with insignificant (dotted) and significant (solid) phase locking (Rayleigh's test $p<0.05$). Insets show distributions of peak phase preferences for units.

H Relationship between strengths of phase locking to 4-5Hz and theta LFP oscillations for single units which showed significant phase preference for both rhythms.

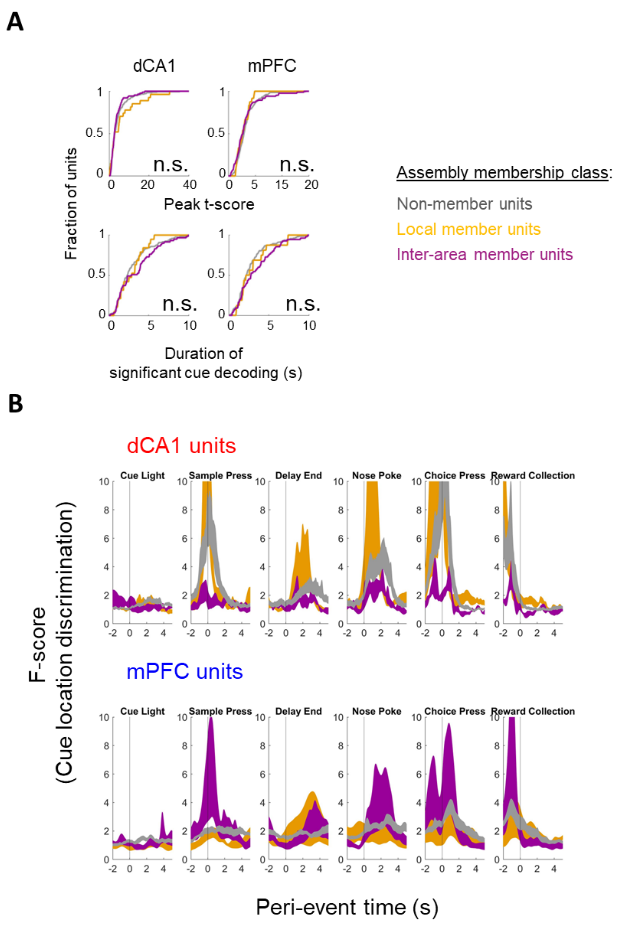

Figure S6: Assembly membership influences population but not single cell properties (relating to Figure 6).

A Classifying dCA1 (left) and mPFC (right) single units as non-members, local members or inter-area cell assembly members did not significantly affect cumulative distributions of peak strength (top) or duration (bottom) of significant cue encoding in the DNMTS task (Kruskal-Wallis ANOVA, Bonferroni-corrected $p>0.05$).
B Evolution of cue discrimination during the DNMTS task by populations of dCA1 and mPFC units is determined by cell assembly membership participation. Time-aligned multivariate cue discrimination (regularised F-scores) of populations of each type of units for each event in the DNMTS task. Shaded regions indicate mean±SEM F-scores for units of each membership classification, from the 12 recording sessions (see methods for details).

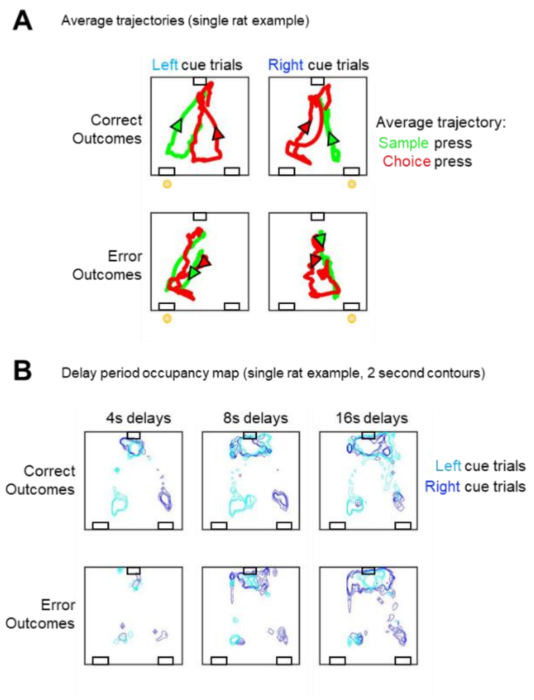

Figure S7: Details of behavior during errors (relating to Figure 7).

A Average spatial trajectories surrounding (±4s) sample and choice lever press events for one example rat on correct and error trials
B Cumulative dwell time (spatial occupancy) of example rat during delays in the DNMTS task on correct and error trials.
