# Supplementary material for: Distinct hippocampal-prefrontal neural assemblies coordinate memory encoding, maintenance, and recall
Source: Curr Biol. 2023 Apr 10;33(7):1220–1236.e4. doi: 10.1016/j.cub.2023.02.029 (PMC10728550; doi:10.1016/j.cub.2023.02.029)
Supplement: Document S2. Article plus supplemental information [file mmc2.pdf]

Current Biology

Article

Distinct hippocampal-prefrontal neural assemblies coordinate memory encoding, maintenance, and recall

Graphical abstract

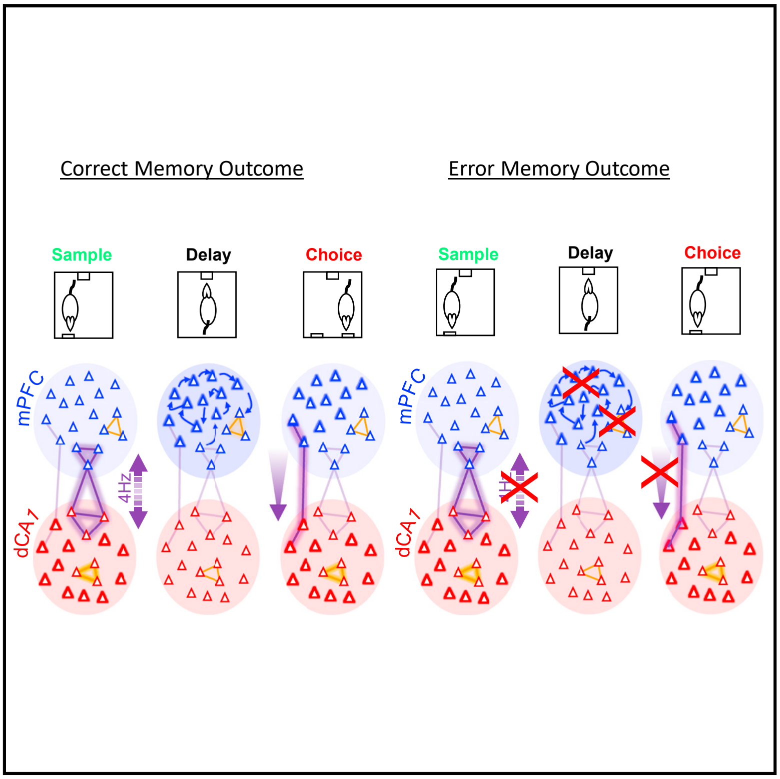

Authors

Aleksander P.F. Domanski, Michal T. Kucewicz, Eleonora Russo, Mark D. Tricklebank, Emma S.J. Robinson, Daniel Durstewitz, Matt W. Jones

Correspondence

michal.kucewicz@pg.edu.pl

In brief

Domanski, Kucewicz, et al. uncover neurophysiological mechanisms that support progression through encoding, maintenance, and recall of memory. Their findings reconcile primate and rodent literatures, identifying 5-Hz rhythmic co-firing of neurons as a signature of assembly dynamics underpinning limbic-cortical interactions during short-term memory.

Highlights

- Hippocampal-cortical (CA1-PFC) activity reconfigures during different memory stages
- Distributed CA1-PFC assemblies co-fire with 5 Hz rhythmicity during memory loading
- Tiled activation in PFC maintains memory across delay periods
- Collapse of rhythmic CA1-PFC assemblies heralds unstable delay coding and errors

Domanski et al., 2023, Current Biology *33*, 1220–1236
April 10, 2023 © 2023 The Authors. Published by Elsevier Inc.
https://doi.org/10.1016/j.cub.2023.02.029

CellPress

Current Biology

Article

Distinct hippocampal-prefrontal neural assemblies coordinate memory encoding, maintenance, and recall

Aleksander P.F. Domanski,[1,6,7,8] Michal T. Kucewicz,[1,2,8,10,11,*] Eleonora Russo,[3,4] Mark D. Tricklebank,[5] Emma S.J. Robinson,[1] Daniel Durstewitz,[3,9] and Matt W. Jones[1,9]
[1]School of Physiology, Pharmacology & Neuroscience, Faculty of Life Sciences, University of Bristol, University Walk, Bristol BS8 1TD, UK
[2]BioTechMed Center, Brain & Mind Electrophysiology Laboratory, Multimedia Systems Department, Faculty of Electronics, Telecommunications and Informatics, Gdansk University of Technology, 80-233 Gdansk, Poland
[3]Department of Theoretical Neuroscience, Central Institute of Mental Health, Medical Faculty Mannheim, Heidelberg University, 68159 Mannheim, Germany
[4]Department of Psychiatry and Psychotherapy, University Medical Center, Johannes Gutenberg University, 55131 Mainz, Germany
[5]Centre for Neuroimaging Science, King's College London, Denmark Hill, London SE5 8AF, UK
[6]The Alan Turing Institute, British Library, 96 Euston Rd, London, UK
[7]The Francis Crick Institute, 1 Midland Road, London, UK
[8]These authors contributed equally
[9]These authors contributed equally
[10]Twitter handle: @BrainMindLab
[11]Lead contact
*Correspondence: michal.kucewicz@pg.edu.pl
https://doi.org/10.1016/j.cub.2023.02.029

SUMMARY

Short-term memory enables incorporation of recent experience into subsequent decision-making. This processing recruits both the prefrontal cortex and hippocampus, where neurons encode task cues, rules, and outcomes. However, precisely which information is carried when, and by which neurons, remains unclear. Using population decoding of activity in rat medial prefrontal cortex (mPFC) and dorsal hippocampal CA1, we confirm that mPFC populations lead in maintaining sample information across delays of an operant non-match to sample task, despite individual neurons firing only transiently. During sample encoding, distinct mPFC subpopulations joined distributed CA1-mPFC cell assemblies hallmarked by 4–5 Hz rhythmic modulation; CA1-mPFC assemblies re-emerged during choice episodes but were not 4–5 Hz modulated. Delay-dependent errors arose when attenuated rhythmic assembly activity heralded collapse of sustained mPFC encoding. Our results map component processes of memory-guided decisions onto heterogeneous CA1-mPFC subpopulations and the dynamics of physiologically distinct, distributed cell assemblies.

INTRODUCTION

Decisions informed by memories of recent experiences are a cornerstone of adaptive behavior and can be modeled experimentally using delayed match or delayed non-match to sample (DNMTS) paradigms. These paradigms require currently relevant information (for example, the location of a transiently presented sample lever) to be (1) loaded into a temporary maintenance buffer, (2) maintained throughout a delay, and (3) integrated with current task rules to inform a choice (e.g., press the opposite lever, not the one presented during sample). Short-term memory's capacity to bridge sample information to context-dependent choice is central to flexible cognition of this type,[1] which is sub-served by interactions spanning executive and mnemonic hub regions including the prefrontal cortex (PFC) and hippocampus.[2,3]

At the cellular level, PFC principal neuron spike rates during delayed response tasks encode diverse features of sample identity and task rules in both non-human primates[4–8] and rodents.[9–15] In particular, sustained PFC principal neuron firing during task delay phases offers an intuitive neural correlate of short-term memory maintenance, bridging sample presentation to choice.[16–32]

However, extending from individual neurons to simultaneously recorded populations has unveiled other informative features of PFC ensemble dynamics.[33–35] For example, sequentially active neurons can "tile" the progression from sample to choice,[24,36–39] and recent models invoke dynamic changes to the information coded by neurons across sample, delay, and response epochs.[20,37,40–47] Such dynamic coding means that the task features encoded by individual neurons can vary across sample, delay, and choice epochs[35,48]; hence neurons not classically selective for individual task features may transiently contribute to short-term memory encoding and maintenance.[49–52] These observations highlight a coding regime that extends beyond straightforward mapping between behavior and the task-selective firing of individual units in PFC.

 © 2023 The Authors. Published by Elsevier Inc.
This is an open access article under the CC BY license (http://creativecommons.org/licenses/by/4.0/).

CellPress
OPEN ACCESS

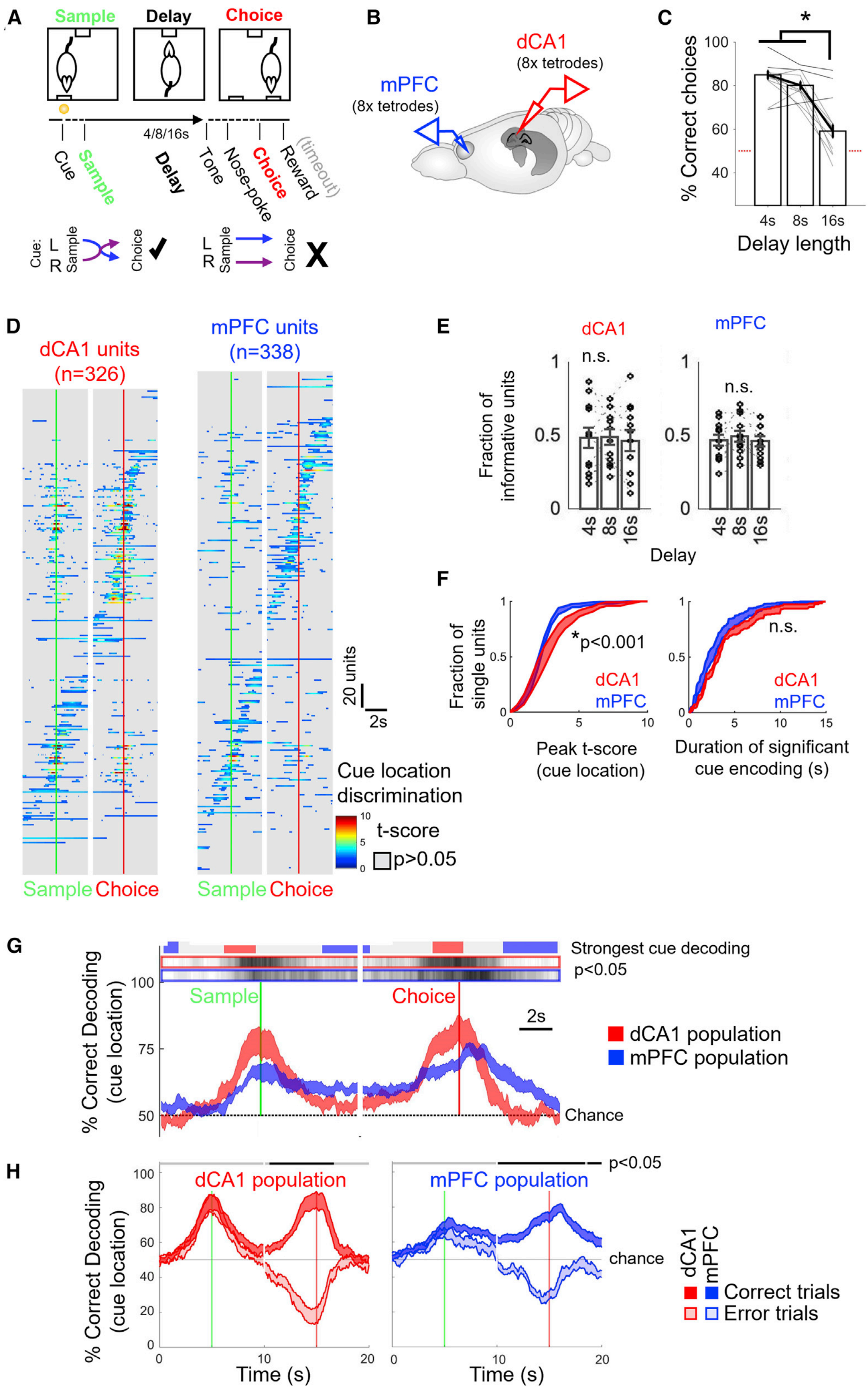

Figure 1. Differential contributions of dCA1 and mPFC neurons and populations to performance in the DNMTS short-term memory task.
(A) Schematic of the DNMTS task (top) and contingencies (bottom). Incorrect choices led to a time-out before the subsequent trial.
(B) Simultaneous hippocampal-prefrontal recording configuration.

(legend continued on next page)

PFC dynamics during delay-dependent short-term memory may hinge, in part, on hippocampal-cortical interactions. Hippocampal CA1/CA3 single unit activity during DNMTS-related tasks in both macaque[53] and rat[11,54,55] shows dissociable sample, delay, and choice correlates. These related patterns of hippocampal and cortical activity are consistent with distributed hippocampal-prefrontal information processing observed in human imaging and electrophysiological studies.[56–62] Indeed, simultaneous recordings from rodent PFC and hippocampus during maze-based non-matching tasks reveal co-varying network activity associated with 5–10 Hz "theta" frequency coherence across the two regions during memory-dependent choice[63–66] and object memory retrieval.[67] Projection-selective optogenetic silencing confirmed that ventral CA1 input to mouse PFC was critical during the sample phase of a T-maze alternation task,[68] while mediodorsal thalamic input to PFC supported the maintenance of information during the delay phase.[24] However, the network dynamics of these interactions that support and distinguish sample, delay, and choice phases remain equivocal.

We set out to disentangle the dynamic contributions of hippocampal and PFC neural assemblies to information encoding during a DNMTS task proven to rely on PFC integrity.[69] We test the hypotheses that (1) correlated groups of neurons distributed across hippocampus and PFC collectively contribute to the optimal representation of cue information during sample encoding and recall, (2) dissociable subsets of PFC neurons (less directly modulated by hippocampus) maintain cue information during the short-term memory delay, and (3) at least one of these population signatures should fail to encode, maintain, or transfer information during errors, culminating in an incorrect choice.

RESULTS

Dissociable hippocampal and prefrontal population dynamics reflect differential contributions to information encoding, maintenance, and recall

We trained six rats on a DNMTS task over 21 days, until their performance averaged 80% correct responses per session at each training stage (Figures 1A, S1A, and S1B; see STAR Methods for task details). Following initial training, we chronically implanted tetrodes (Figures 1B and S1C–S1F) to record simultaneous spiking activity from dorsal CA1 and prelimbic medial prefrontal cortex (mPFC); data are presented from the final two DNMTS sessions after criterion had been achieved (STAR Methods). After spike sorting and thresholding for mean firing rate >0.5 Hz during the task, 31 ± 5 mPFC and 30 ± 5 dCA1 (mean ± standard error of the mean [SEM]) well-isolated putative principal neurons were analyzed per session.

Rats made significantly more errors on 16 s delay trials than trials with shorter delays (Figure 1C, N = 12, 2 sessions from 6 rats, ANOVA, $F(2,36) = 42.4$, $p < 0.001$, Tukey-Kramer post-hoc test for delays $p < 0.001$): all rats performed significantly above chance on 4 and 8 s delay trials ($p < 0.05$, binomial tests for each rat's performance), whereas only three rats achieved above-chance performance at 16 s delay. Latencies between cue, sample lever press, end-of-delay nosepoke and choice lever press did not vary systematically with delay, or correct vs. error trials (Figure S1B), meaning that inaccurate performance was unlikely to stem from failure to engage with the task.

To quantify the time-varying encoding of left and right cue location by single units, we used Student's t test as a measure of discrimination between left- vs. right-trial firing rates in 50ms bins for each neuron's trial-averaged activity in dCA1 and mPFC (Figure 1D). For dCA1 units, left- vs. right-trial discrimination tended to peak around sample and/or choice lever presentations. The activity of mPFC units was less bound to lever presses, sequentially tiling the entire delay period (Figure 1D, bottom right panel and Figures S1H–S1J). Approximately half (dCA1: 48%, mPFC: 57%) of units were informative (provided significant left vs. right information for >50 ms within ±4 s of the sample or choice lever presses), with fractions of informative units consistent across delays (Figure 1E, Kruskal-Wallis ANOVA; dCA1: $H(2) = 0.18$, $p = 0.91$; mPFC: $H(2) = 0.44$, $p = 0.80$). However, while dCA1 units showed significantly stronger peak cue location encoding than mPFC units during the sample and choicepreparatory periods (Figure 1F left: peak t-score, dCA1 vs. mPFC units; 3.49 ± 0.23 vs. 2.72 ± 0.08, $t(463) = 3.52$, $p = 0.00048$, t test, N = 223, 242 units from 12 sessions), single units from the two areas were indistinguishable in the durations over which they encoded cue location (Figure 1F, right: duration of encoding, dCA1 vs. mPFC units; 2.24 ± 0.19 s vs. 1.99 ± 0.16 s; $t(463) = 1.38$, $p = 0.17$, t test, n = 223, 242 units from 12 sessions). Very few mPFC units showed persistent lever-selective delay firing (approximately 85% units showed significant decoding for <6 s, Figure 1F, right). Taken together,

(C) Performance of 6 rats (2 sessions from each) across delay lengths. Black dotted/solid lines link trials from at/above-chance sessions; red dotted line indicates chance. All rats performed above chance for 4–8 s delays, but only 3 out of 12 for 16 s delay trials.

(D) t scores between left/right sample trial firing rates; units aggregated across sessions and sorted by times of peak discrimination. Gray regions mask periods of insignificant cue location discrimination ($p > 0.05$, against bootstrapped 95% CIs).

(E) Fractions of units recorded in each session providing significant left/right decoding (bootstrapped Bonferroni-corrected $p < 0.05$) for >50 ms, sorted by delay length. No significant differences were observed across delays for either area.

(F) Distributions of peak strength (left) and duration (right) of left/right encoding by dCA1 and mPFC units. Shaded regions indicate mean ± SEM of distributions across sessions.

(G) Leave-one-out decoding of left/right-trial type from firing rates of single units. Shaded curves indicate mean ± SEM decoding across animals with matched trial and unit counts randomly sampled from available data. Gray shaded bars above indicate periods of cue decoding significantly different from chance (cue-shuffled data). Blue/red bars show periods of significantly stronger cue decoding from mPFC/dCA1 units ($p < 0.05$, bootstrap permutation test between the two conditions N = 12 subject sessions).

(H) Performance of regularized linear decoder trained on correct trials and tested on correct (solid) or error trials (transparent). Mean ± SEM performance across recording sessions shown. Black bars indicate times of significant drops in cross-validated decoding performance during errors (bootstrap permutation test, Bonferroni-corrected $p < 0.05$).

See also Figure S1.

CellPress
OPEN ACCESS

these observations corroborate evidence that short-term memory can be supported by populations of transiently activating neurons.[24,46,70,71]

We next considered how joint activity of simultaneously recorded populations of single units within each area contributed task-relevant information. To directly compare sample lever coding between the two populations, we employed a linear discriminant classifier based on vector representations of single neuron instantaneous firing rates in 50-ms bins (Figure 1G). We included single units with individually significant cue location encoding for >50 ms (as in Figure 1E) in this and subsequent analyses. Each trial of the task was omitted in turn and a linear classifier trained on the remaining trials (leave-one-out cross-validation, LOOCV) to predict the class label (left vs. right sample lever) of the withheld trial. To compare between animals, random subsets of equalized unit numbers and trial counts were drawn between conditions to rule out dimensionality confounds in classifier performance.

dCA1 populations showed strong but transient readout of sample position, which peaked around the lever presses and dropped to chance decoding performance during the delay period (Figure 1G, red traces). The discrimination around sample and choice lever presentation was less pronounced in mPFC populations, which instead maintained a stronger representation of sample identity than dCA1 populations throughout the delay and post-choice evaluation period during reward consumption (Figure 1G, blue traces). These findings are in good agreement with recent comparisons between task coding dynamics in hippocampal and frontal cortical populations in primates.[72,73]

Which features of the dCA1 and mPFC population activity are essential to the correct execution of the DNMTS task? Previous studies have induced forced errors, by lesioning or inactivating targeted parts of the hippocampal-frontal network,[65,68] but less is known about the system's behavior during spontaneous, unforced errors. Since 16 s delay trials challenged the short-term memory limits of rats, we quantified which aspects of the sequential contributions of dCA1 and mPFC populations failed during incorrect choices.

Decoders trained on correct trials and tested on error trials demonstrated that decoding of sample position from hippocampal populations was intact (Figure 1H): the two conditions were indistinguishable around the sample lever press but, on error trials, dCA1 represented the wrong (opposite) position on approach to the choice lever press. Thus, even as the rats revisited the sample (wrong) location, hippocampal representations remained faithful. In the mPFC, however, sample location representation began to decay immediately after the sample lever press on error trials, such that incorrect choices could be predicted approximately 2 s earlier in the delay period than from dCA1 activity (black bars in Figure 1H indicate significantly error-predicting periods).

mPFC population dynamics support coding that spans the DNMTS delay phase

To test which ensemble mechanisms might underlie stable coding by mPFC populations during the delay period, we assessed trial-by-trial associations between mPFC population dynamics and DNMTS accuracy (Figure 2). Since 4, 8, and 16 s delay trials were presented to the rats in randomized order, we first sorted trials by delay length to compare LOOCV decoding across delays and correct vs. error outcomes. Subsets of the different trial types were drawn at random to allow matching of trial numbers across conditions (accounting for fewer available correct trials on longer delays).

On 4 s and 8 s delay trials, delay period decoder results were significantly better than chance (bootstraps with shuffled trial labels) for the entire delay period duration in the majority of recording sessions (indicated by gray shading in Figure 2A). On average, rats performed at chance during 16 s delay trials, i.e., the equivalent of guessing randomly. This means "correct" responses may have arisen from lucky guesses, independent of CA1-mPFC information processing. We therefore split recording sessions by whether behavioral performance was at, or significantly above, chance performance during that session (light and solid blue curves in Figure 2A). Whereas correct outcome 16 s delay trials from above-chance sessions showed decoder performance that was above chance for the majority of the delay period (albeit stronger during the first 8 s and variable due to the small subset of the above-chance sessions), mPFC population decoding from correct trials from chance-performance behavioral sessions fluctuated around chance levels from shortly after sample lever press. Thus, even though all trials examined corresponded to "correct" outcomes, faithful decoding of cue identity from mPFC populations depended on whether the rats were performing the task better than chance as opposed to guessing, implicating faithful cue representation by mPFC populations in successful task performance.

What form does the delay-spanning coding scheme take in mPFC? One possibility is that firing rates across neurons evolve in fixed proportions relative to one another, such that a decoder trained on population firing rates at the start of the delay successfully predicts left vs. right sample lever identity using firing rates from the end of the delay, and vice versa. Alternatively, a dynamic code implemented by the mPFC population may mean decoding results are only transiently valid around the time of the training data. We compared evidence for these two hypothetical schemes by constructing decoders using population firing rate data from each 50 ms segment of the delay period and systematically "sliding" the test data across the entire delay period (Figure 2B, method reviewed in Meyers et al.[74]) These results form symmetrical cross-temporal decoders in which the diagonal (white arrows in Figure 2B) represents training and testing performed at matching time points.

For 4 and 8 s delay trials, decoders trained and tested at any time during the delay period were similarly valid, as indicated by the extended region of significant off-diagonal decoding performance throughout the delay (marked by white bounding boxes in Figure 2B). During 16 s delay trials in sessions with above-chance behavior, a window of significant cross-temporal decoding (skewed rightwards in Figure 2B) revealed that the mPFC population can reliably encode the location of the initial cue during the whole delay period. This sustained representation was not seen in dCA1 (Figure S2), or in decoding results from sessions with at-chance behavioral performance (Figure 2C). Instead, despite an initial early-delay period of transient decoding comparable in strength to the above-chance sessions (as in Figure 2B), cross-temporal decoding did not outlast approximately 2 s. These results implicate strength of readout of cue

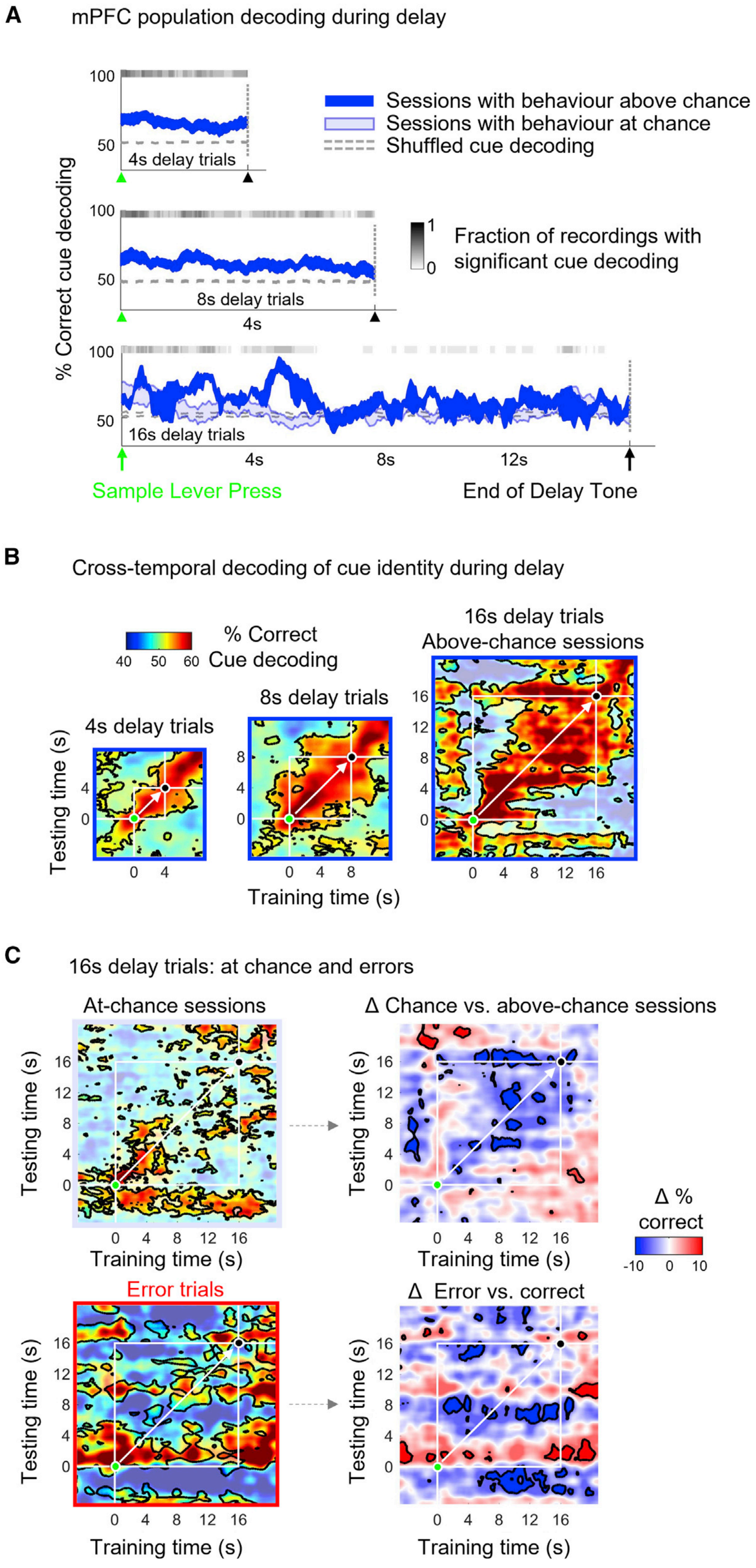

Figure 2. Maintenance of cue location by a stable population code in mPFC underlies correct performance in the DNMTS task.

(A) Time-resolved decoding of cue location from mPFC single-unit populations on correct trials for sessions above (solid blue) and below (light blue) chance performance (50%, dotted line). Gray bars above curves show fraction of above-chance performing sessions with significant decoding. Mean ± SEM decoding performance from 12 sessions (all above-chance performance for 4 and 8 s delays, nine at chance for 16 s delay trials).

(B) Cross-temporal decoding during the delay period: cross-validated regularized linear decoders trained and tested at different time points during the delay period (±5 s). White lines indicate bounding times of delay period. Green and black markers indicate sample press and end-of-delay tone, respectively. White arrow along diagonal indicates training and testing at the same time point (using different withheld trials for testing), recapitulating curves shown in (A). Bounded regions show significant ($p < 0.05$) decoding relative to cue-shuffled bootstrap distribution.

(C) Cross-temporal cue decoding performance of mPFC unit population recordings on correct 16 s delay trials from at-chance sessions (top) and on errors on above-chance (bottom). Statistics as for (B). Right: subtraction of above-chance session decoding from below-chance sessions (top) and correct from error trials (bottom). Bounded regions indicate significant differences between conditions (bootstrap permutation test, $p < 0.05$).

See also Figure S2.

identity from mPFC populations in supporting successful DNMTS task performance. Consistent with this interpretation, the sustained mPFC population code evident during correct 16 s trials failed during the at-chance and the error trials (Figure 2C, lower panels).

A sparse subset of neurons form joint dCA1-mPFC cell assemblies optimally encoding cue information during the DNMTS task

Given the encoding of dissociable task-related information in dCA1 and mPFC populations, when and how is information shared between the two regions? Correct activation of the dCA1-mPFC pathway is essential for performance of spatial short-term memory tasks[68,75,76] and presumably must induce systematically covariant inter-regional activity at some point(s) during delayed responding.

We developed a cross-validated factor analysis method (FA[77,78]) to detect coordinated activity among units from mPFC, dCA1, or jointly from mPFC and dCA1 (Figures 3 and S3; STAR Methods). FA is a model-based statistical tool that explicitly captures correlations between variables through a set of independent factors (and assuming independent noise sources). FA has been shown to outperform principal-component analysis (PCA) for the purposes of dimension reduction, neural manifold reconstruction, and cell assembly detection[78,79] and has recently been used to probe neural population activities underlying decision-making in rodent frontal cortex.[80]

dCA1 and mPFC units were assigned to the same cell assembly when they significantly loaded on the same latent factor (Figures 3B and 3C), where latent factors captured correlated firing rate activities within the population of neurons (Figure 3D). We detected significant inter-area cell assemblies in four of six rats. FA-detected assemblies were in good agreement with those detected with a previously established PCA-independent component analysis (ICA) method[81] (Figure S3A). Inter-regional assemblies were more numerous (Figure S3B) and larger (Figure S3C) than within-region assemblies but comprised a sparse minority of total recorded units (Figure S3D). Cell assemblies were largely non-overlapping (Figure S3E) and not biased by the mean firing rates of their constituent neurons (Figures S3F and S3G). The time-varying factor scores derived from the FA model (Figure 3B) can be taken as a measure of assembly activation strength, as exemplified by significant event-locked activation of inter-area assemblies linking dCA1 and mPFC units during the DNMTS task (Figures 3D, S3H, and S3I).

Assemblies inherited the cue encoding properties of their member units. In the case of inter-regional assemblies, cue decoding analysis revealed combinations of each area's encoding features such as the strong encoding around lever presses of dCA1 members, and pre-choice encoding of mPFC members (Figures 4A and S4A). Assemblies detected within dCA1 provided better peak cue encoding than mPFC assemblies, on average, as was observed with single unit encoding (Figure 1F). However, encoding strengths of inter-regional assemblies were indistinguishable from individual area local assemblies, consistent with a mixing of cue encoding features from both areas (Figure 4B). Thus, FA-detected cell assemblies were typically small and sparse but provided better cue encoding than their member units (Figure 4C). Overall, more dCA1 and inter-area cell assemblies were informative for >50 ms during the task than their member units, with a similar trend observed for mPFC assemblies (Figure 4D).

We next probed the optimality of units' arrangement into cell assemblies. Intuitively, the correlated firing of similarly-tuned neurons spanning dCA1 and mPFC networks could provide increased robustness to trial-to-trial firing rate variability of individual neurons.[82] Conversely, mixing of complementary information from dCA1 and mPFC neurons providing cue encoding at staggered times could increase the duration over which information readout is possible from the inter-area assembly.

Grouping neurons by similarity of activity (Figure S4B) suggests that cue encoding is improved by cell assembly formation via boosting signal correlations between members. To test how many units are required to support this coding enhancement, we used established methods to create synthetic cell assemblies with sizes matching FA-detected neurons[49,83,84] and optimized to include the best possible combinations of units aggregated according to their ranked peak cue information (Figure 4E). We compared the peak cue decoding of the assemblies detected by FA against that of size-matched synthetic cell assemblies drawn from all available units in that recording, matching the real cell assemblies to the closest ranked synthetic assembly (Figure 4F, expanded in Figure S4C). Peak cue decoding performance rose rapidly with increasing assembly size and was near optimal (approaching 100%, exceeding the best or second-best unit combination of synthetic assemblies) with group sizes of <5 units, comparable to those observed in the FA-detected assembly pool (Figure S3C). Inter-area assemblies were within the top two best possible combinations of dCA1 and mPFC units and reflected better decoding than by mPFC units alone (Figure 4G). Therefore, across all FA-detected assemblies, performance was skewed toward optimally representing cue location; however, integration of correlated dCA1 and mPFC activity boosts the cue representation over that in mPFC alone.

Together these results reveal that sparse groups of neurons spanning the dCA1-mPFC network form cell assemblies which, by averaging noisy firing rate fluctuations of single neurons and boosting signal robustness, enhance the encoding of cue information over that of single units alone and form near optimal representations within small groups (<10 units).

Rhythmic firing hallmarks joint dCA1-mPFC cell assembly dynamics and memory performance

We found that units that formed dCA1-mPFC assemblies (Figures 3D and 5A) showed 4–5 Hz rhythmic modulation in their spike train autocorrelations (Figure 5B). dCA1-mPFC unit pairs drawn from assemblies showed coherent spike train modulation at 4–5 Hz, which was weaker for pairs drawn from different cell assemblies, and weaker again for pairs of units not detected as cell assembly members (Figure 5C: average 3.5–5.5 Hz coherence across dCA1-mPFC cell pairs, $F(2,27) = 50.0$ $p < 0.0001$, Tukey-Kramer post-hoc tests for assembly membership $p < 0.05$, $N = 2$ sessions from 6 animals). Coherent 4–5 Hz spike train modulation was also weaker for pairs drawn from within-area cell assemblies in dCA1, and essentially absent between pairs from mPFC. This indicates that, on average, "non-assembly" units were physiologically distinct from their assembly

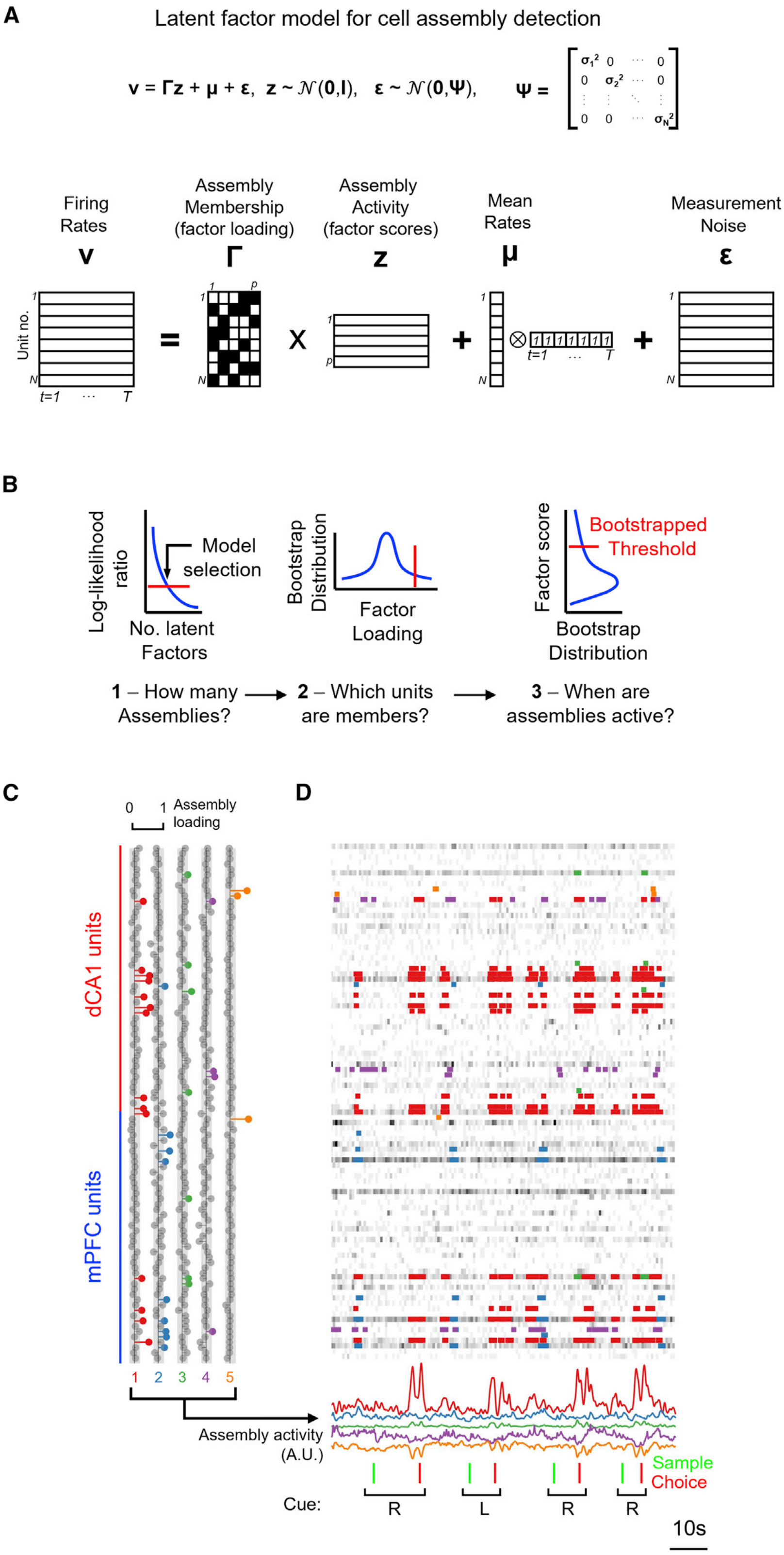

Figure 3. A latent factor analysis model detects correlated inter-regional dCA1-mPFC cell assemblies.

(A) Schematic of the FA-based cell assembly detection, decomposing parallel recordings from N single units into time-varying activation scores of p ($p < N$) factor scores.

(B) Model selection steps involved in FA-based cell assembly detection.

(C) Inter-area cell assembly detected from dCA1-mPFC recording. Example loading of single units to the five detected latent factors (cell assemblies). Gray circles indicate units with insignificant loading strengths (shaded columns indicate $p < 0.01$ vs. shuffled bootstrap distributions).

(D) Example spike rasters from units in (C) during four successive trials of the DNMTS task. Color annotation on spike trains indicates times of significant activation (bootstrap $p < 0.01$, vs. factor model calculated from shuffled spike rates) for each detected dCA1-mPFC assembly.

See also Figure S3.

CellPress
OPEN ACCESS

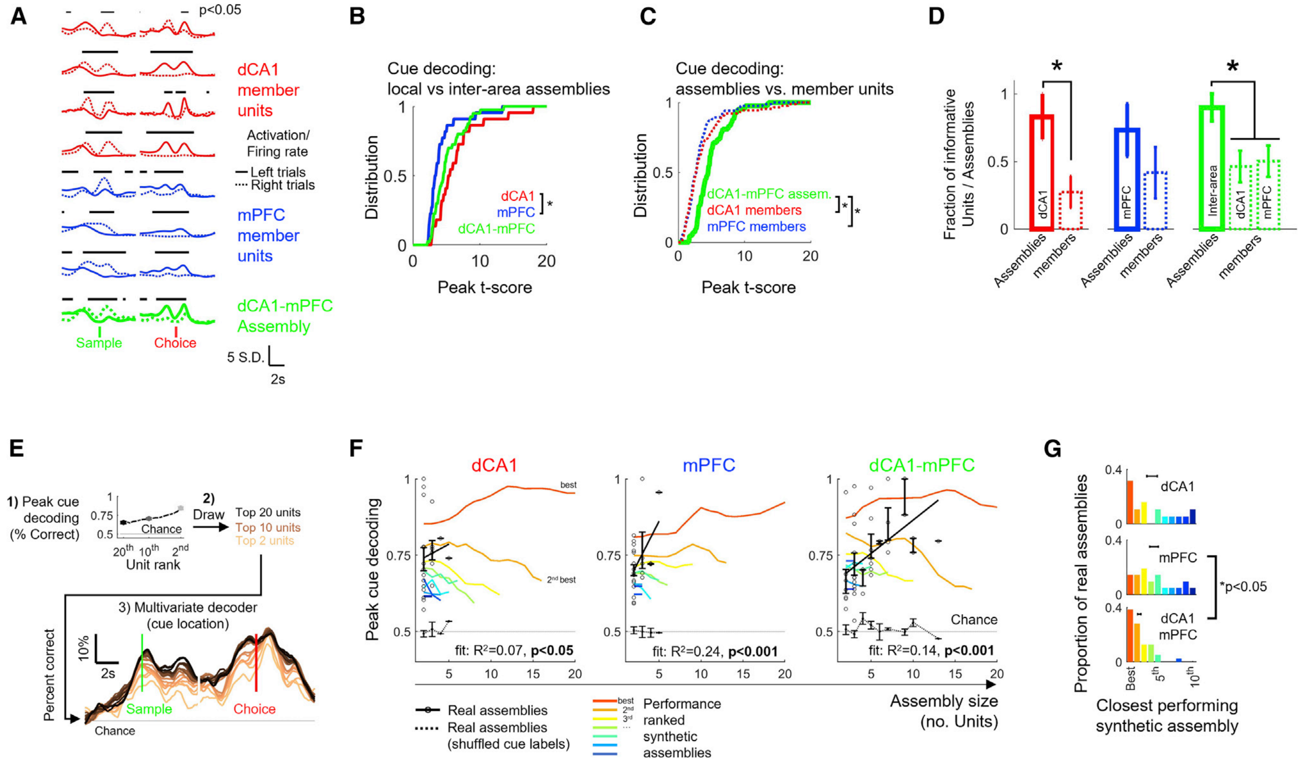

Figure 4. A sparse subset of neurons form joint dCA1-mPFC cell assemblies optimally encoding cue information during the DNMTS task.

(A) Trial-averaged dCA1-mPFC assembly activity (green) and spike rates of dCA1 and mPFC member units (red and blue, respectively) on correct left and right trials. Same example as Assembly 1 in Figures 3C and 3D (red annotation). Times of significant cue location encoding (bootstrap Bonferroni-corrected $p < 0.05$) indicated by black bars.

(B) Distribution of peak cue decoding strength from each class of cell assembly (Kruskal-Wallis test: $X^2(2,81) = 10.59$, $p = 0.005$). Asterisk: Tukey-Kramer post-hoc test, $p < 0.05$.

(C) Distributions of cue location decoding of dCA1-mPFC cell assemblies (green) vs. constituent single units (broken lines). Kruskal-Wallis test was used: $X^2(2,213) = 23.98$, $p < 0.01$. Asterisks indicate Tukey-Kramer post-hoc tests with comparisons ($p < 0.05$).

(D) Fractions of cell assemblies (solid bars) and constituent single unit members (broken bars) providing significant cue location decoding for $\geq$ 50 ms. Asterisks indicate significant differences between assemblies and units for each assembly type (Mann-Whitney U test: p = 0.0127/p = 0.316/p = 0.033 for CA1/mPFC/dCA1-mPFC, respectively).

(E) Testing left/right sample decoding capacity from synthetic cell assemblies. (1) Single units are ranked from best to worst by individual peak strength of sample location decoding. (2) Rank-ordered sequential draws of size 2–20 are chosen to provide groups of units (e.g., for the top two assembly draws of size of 3: take units in rank position [1, 2, 3], [4, 5, 6], etc.). (3) Leave-one-out multivariate decoding of cue location from each combination of {assembly size, rank order} was used for peak decoding performance preceding choice press measured.

(F) Cue location decoding performance of rank-ordered optimal synthetic groups (colored lines) vs. units forming FA-detected cell assemblies (black points). Error-bars indicate SEM of peak decoding for assemblies of a given size. Dotted lines indicate decoding from FA-detected assemblies with shuffled cue location labels. Black lines are best linear fits to peak decoding vs. assembly size for FA-detected cell assembly member decoding.

(G) Ranks of closest-performing synthetic cell assemblies for each FA-detected cell assembly grouping. Horizontal bars show mean ± SEM rank. Asterisk indicates Kruskal-Wallis test between assembly type: $X^2(2,76) = 9.99$, $p = 0.0068$.

See also Figure S4.

counterparts. The 4–5 Hz assembly motif was specific to the task period and absent from spike trains recorded during 1 h rest periods flanking DNMTS sessions (Figure S5A).

We wondered whether the parsing of the two crucial "sample" and "choice" events of task context might be visible in the patterned firing of the assembly member units. Rhythmic dCA1-mPFC coactivity did indeed emerge between pairs of cells in a task phase-dependent manner, with 4–5 Hz modulation most prominent during the 4 s preceding sample lever presses (Figures 5D and S5B). This rhythmic signature of dCA1-mPFC assembly activity therefore timestamped the DNMTS sample phase and is consistent with evidence that optogenetic silencing of hippocampal-prefrontal interactions is particularly disruptive during the sample phase of delayed responding on a T-maze.[68]

dCA1-mPFC assembly member pairs showed additional temporal structure at slower timescales: cross-correlations preceding choice lever presses were not 4–5 Hz modulated but tended to reflect mPFC spiking leading dCA1 spiking (Figure 5D, bottom right panel), indicating a shift in the direction of signal flow between hippocampus and frontal cortex on transition from sample to choice. Such a context-dependent shift has been suggested in previous analyses of decision-making.[67,85,86] We further explored this shift by examining when individual assemblies

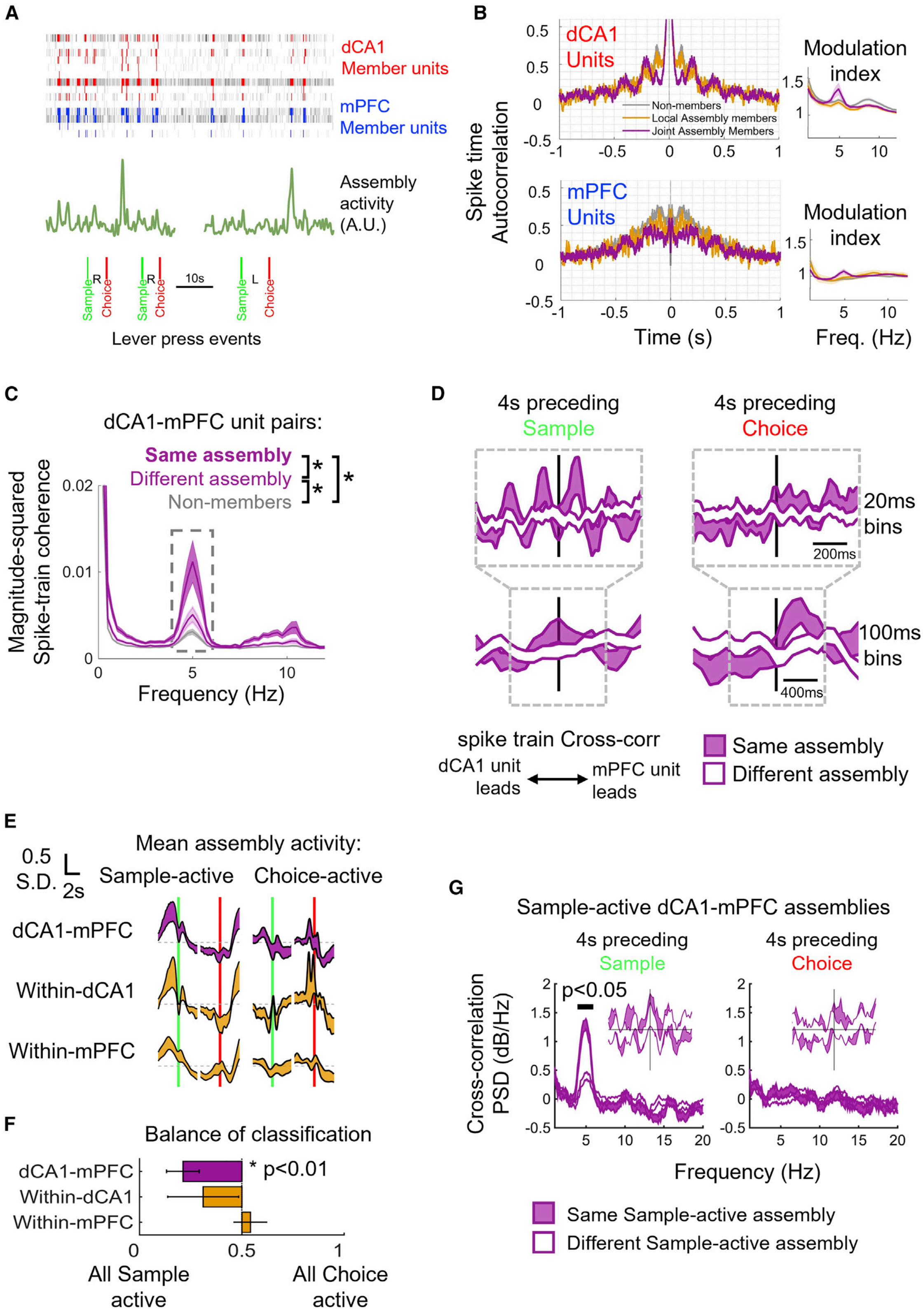

Figure 5. Distinct rhythmic firing signatures hallmark joint dCA1-mPFC cell assembly membership during the DNMTS task.
(A) Spike trains of units comprising assembly 1 (red annotation in Figures 3C and 3D) highlighted to show firing of dCA1 and mPFC (red and blue, respectively) units during significant assembly activation times.

(legend continued on next page)

were most active during sample or choice events. Figure 5E summarizes the time-varying activities of within-area (gold) and inter-area (purple) cell assembly activities during the task, demonstrating comparable activation during sample presentation but diverging activation levels during delay and choice events. Taking assemblies with factor scores showing significant activity in the time preceding lever presses, we classified them as either "sample-active" or "choice-active" based on the time of their strongest average activation (Figures 5F and S5B). Both classes of sample and choice activity were approximately equally represented in within-mPFC assemblies (9 sample-active vs. 10 choice-active detected assemblies). Conversely, a significant majority of within-dCA1 and dCA1-mPFC assemblies were sample active (dCA1: 13 sample-active vs. 5 choice-active; dCA1-mPFC: 20 sample-active vs. 6 choice-active detected assemblies, one-sample t test vs. an even sample/choice split for within-animal averages; mPFC: $T(6) = -2.61$, $p = 0.08$, dCA1: $T(6) = -2.53$, $p = 0.13$, dCA1-mPFC: $T(6) = -6.72$, $p < 0.01$, Figure 4F). This could not be explained by skewed unit counts across areas in our recordings or over-representation of one area's contribution to the FA assembly models (Figures S5C and S5D). Finally, restricting analysis of rhythmic dCA1-mPFC cell pair interactions to assemblies most active during the sample period amplified the 4–5 Hz rhythmic coordination (Figure 5G). The coherent modulation of spike trains by this fingerprint "sample" rhythm was attenuated between parallel but independent dCA1-mPFC assemblies and was absent in the activities of sample assemblies during the choice-preparatory period.

Our assembly analyses reveal sparse subsets of single units that cohere task-dependently into assemblies spanning dCA1 and mPFC and are hallmarked by a physiological signature of 4–5 Hz coordination. As such, the inter-area cell assemblies are uniquely positioned to orchestrate dCA1-mPFC interactions and information transfer. However, 4–5 Hz rhythmic activity did not manifest in the local field potential (LFP) oscillations. Unlike hippocampal theta (8–12 Hz) oscillations, which were prominent in LFP spectrograms from dCA1 tetrodes during the DNMTS task, no clear rhythmic oscillations in the 4–5 Hz band were observed in either brain area (Figure S5E). Similarly, clear dCA1-mPFC oscillatory LFP coherence was observed in theta but not in the 4–5 Hz bands (Figure S5F).

Subsets of units from dCA1 and mPFC showed significant spike phase-locking with respect to the within-area 4–5 Hz LFP band (39% and 33%, of 198 and 207 units from seven recordings, dCA1 and mPFC, respectively, $p < 0.05$, Rayleigh test of circular uniformity). Of significantly modulated units (solid lines and histograms, Figure S5G, left), modulation strengths were weak and indistinguishable between brain areas ($z = 0.21$, $p = 0.22$, Mann-Whitney U test). In contrast, theta-tuned units were significantly stronger tuned in dCA1 than mPFC (Figure S5G, right, $z = 9.12$, $p < 0.0001$, Mann-Whitney U test), such that individual dCA1 units, which co-tuned for both 4–5 Hz and theta bands, showed on average twice stronger tuning for theta than for 4–5 Hz. Units from mPFC did not show this bias (Figure S5H: theta M.R.L. / 4–5 Hz M.R.L. ratio = 2.3 vs. 1.1 for dCA1 vs. mPFC, respectively; $z = 5.85$, $p < 0.001$, Mann-Whitney U test). These findings confirm the weak influence of the 4–5 Hz LFP modulation on units' spike times and that theta and 4–5 Hz rhythms are distinct from one another.

We next sought to map the activities of assembly neurons onto component processes of short-term memory underlying DNMTS task performance. Examining times at which the three different classes of assembly-participating units (intra-CA1, intra-mPFC, and dCA1-mPFC) provided significant cue encoding during the task did not show clear segregation at the single cell level (Figures 6A and S6A). However, multivariate population decoding from units belonging to these different assembly types did demonstrate dynamic, fluctuating contributions of each neuronal class during the DNMTS task (Figures 6B and S6B). Shaded ticks above the dynamic curves in Figure 6B track qualitatively the time-evolving strongest "winner-take-all" decoding of cue location from each sub-population of units, with the constraint that each area must show significant decoding when considered individually. For example, mPFC inter-area assembly members (purple traces) showed strong sample representation, while non-members better discriminated left vs. right trials during the delay.

These analyses demonstrate a dynamic modulation in the representation of cue information by cells forming assemblies that

(B) Left: rate-normalized spike-time autocorrelations (mean ± SEM across units) for non-assembly units, members of within-area and inter dCA1-mPFC cell assemblies for each area. Right: modulation index of spike train autocorrelation functions.

(C) Inter-area spike train coherence between dCA1-mPFC unit pairs within, across and outside cell assemblies (mean ± SEM of dCA1-mPFC pairs across sessions shown). Gray shaded region indicates frequency range used for statistical comparison of rhythmic modulation (asterisks mark significant difference).

(D) Pairwise dCA1-mPFC spike train cross-correlogram (top: 20 ms bins, bottom: 100 ms bins) for spikes fired by units in the 4 s preceding sample (left) and choice (right) lever press events. Average across all pairs shown (mean ± SEM across recording sessions) for within (purple) and across (white) assembly pairs. Random sampling of spikes (100 draws per cell pair) was used to match firing rate offsets between cells. Cross-correlations are normalized by total spike count.

(E) Standardized activation patterns (factor score activity) of different classes of cell assembly (mean ± SEM averaged across sessions), aligned to sample and choice lever press events. Assemblies were sorted by peak activity time and categorized as either "sample-active" or "choice-active" (left and right columns, respectively). Dashed lines show mean assembly activities for each class.

(F) Fractions of cell assembly categorized as sample- and choice- active for each assembly type detected in the 12 recording sessions. 0 and 1 indicate assemblies most strongly activated during the sample and choice phases respectively. Asterisk indicates that dCA1-mPFC assemblies were significantly biased toward more sample-active.

(G) Rhythmic 4–5 Hz dCA1-mPFC spike correlations (as in D) were specific to pairs of units from sample-active assemblies and were strongest for inter-regional pairs drawn from the same cell assembly.

See also Figure S5.

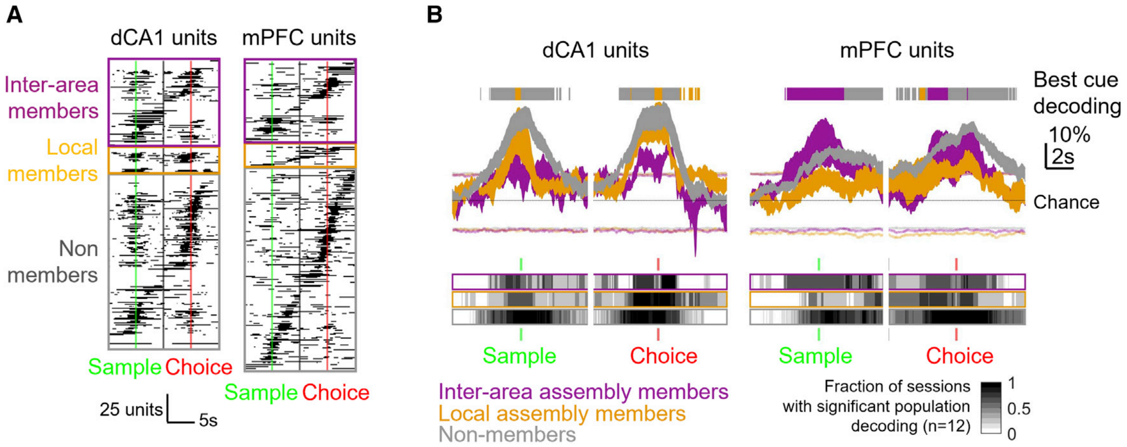

Figure 6. Assembly membership-dependent differences in the statistics of cue encoding during the DNMTS task are not visible at the single neuron level but orchestrate dynamic contributions by neural populations.

(A) Discrimination of cue location by firing rates of individual dCA1 and mPFC single units, sorted by assembly membership type and time of peak discrimination. Black bars indicate time of significant decoding (t test, Bonferroni-adjusted $p < 0.05$, bootstrapped confidence limit).

(B) Population-level discrimination of cue location by firing rates differs by class of assembly membership. Curves show cross-validated multivariate decoding for units participating in different assembly types (mean ± SEM across recording sessions). Colored lines indicate 5%, 95% bootstrap CIs of decoding from shuffled trial labels (mean across session shown). Below: gray shading indicates fraction of recording sessions with decoding exceeding 95% CI at each time point. Above: best performing group provides significant decoding from >60% sessions, winner takes all.

See also Figure S6.

cannot be detected in the activities of individual member neurons. In particular, changes in the pattern of interaction across brain areas hallmark parsing of the cognitive contexts of encoding, maintenance, and recall lever presses during the task.

Incorrect choices are associated with impaired transfer of dCA1 cue information to mPFC with collapse of intra-mPFC dynamics

These distributed and dynamic mechanisms associated with successful completion of the DNMTS task present multiple potential vulnerabilities to disruption, culminating in erroneous choices. Although we could not detect overt changes to behavioral strategies during error trials (Figures S7A and S7B), we did observe several features of coordinated population activity that were altered on error trials (Figure 7). The average activation profiles of within-dCA1 or sample-active within-mPFC assemblies were unaffected on error trials (Figure 7A, top), whereas choice-active, within-mPFC assembly activation was significantly weakened in the period leading up to the choice lever press (gold vs. red traces Figure 7A, third row). No differences were observed in average activation strengths of inter-area dCA1-mPFC assemblies preceding the incorrect choice lever presses, but activation was significantly stronger immediately afterwards (purple vs. red traces in Figure 7A, bottom), suggesting coordinated inter-area firing in error feedback signaling, as has been reported for this pathway in primates.[72]

We wondered whether the rhythmic firing before sample and mPFC-driven correlations before choice times (Figures 5E–5G) would be specifically affected on errors (gray boxes in Figure 7B). Indeed, we observed significantly weaker 4–5 Hz correlated firing between inter-area pairs from the same assembly during the sample-preparatory period (Figure 7C), and a reverse in lag of peak correlation, such that dCA1 led mPFC firing in the choice-preparatory period on error trials, instead of mPFC leading dCA1 as in correct trials (Figure 7D, $t(250) = 2.21$, $p = 0.0274$, paired t test). These altered signatures of CA1-mPFC interaction during unforced errors further implicate rhythmic hippocampal-prefrontal network population coordination during short-term memory loading in later incorrect choice-making.

DISCUSSION

Simultaneous electrophysiological recordings afford an integrated view of dCA1-mPFC information coding and exchange during delayed non-matching behavior. Alongside substantiating the differential hippocampal and prefrontal contributions to DNMTS performance indicated by previous single-region recordings and/or lesioning studies, our approach thereby unveiled three principal findings: (1) the existence of distributed, dCA1-mPFC assemblies, recruited during sample encoding and hallmarked by 4–5 Hz rhythmic co-modulation, (2) delay-dependent maintenance of cue information by a separable subset of mPFC neurons, not bound into dCA1-mPFC assemblies, and (3) failure of dCA1-mPFC assemblies to load short-term memory during unforced errors, with unstable mPFC delay coding culminating in incorrect choices.

Ensemble activity "within" rat dorsal hippocampus[87] and population or cell-pair firing patterns "within" mPFC have previously been associated with behavioral performance[46,70] and hippocampal network oscillations[64,88] during delayed response tasks. However, most prior rodent recordings were made during maze-based tasks, which offer less control over precisely when animals encode information and blur mnemonic and decision-making processes in space and time. The temporal

CellPress
OPEN ACCESS

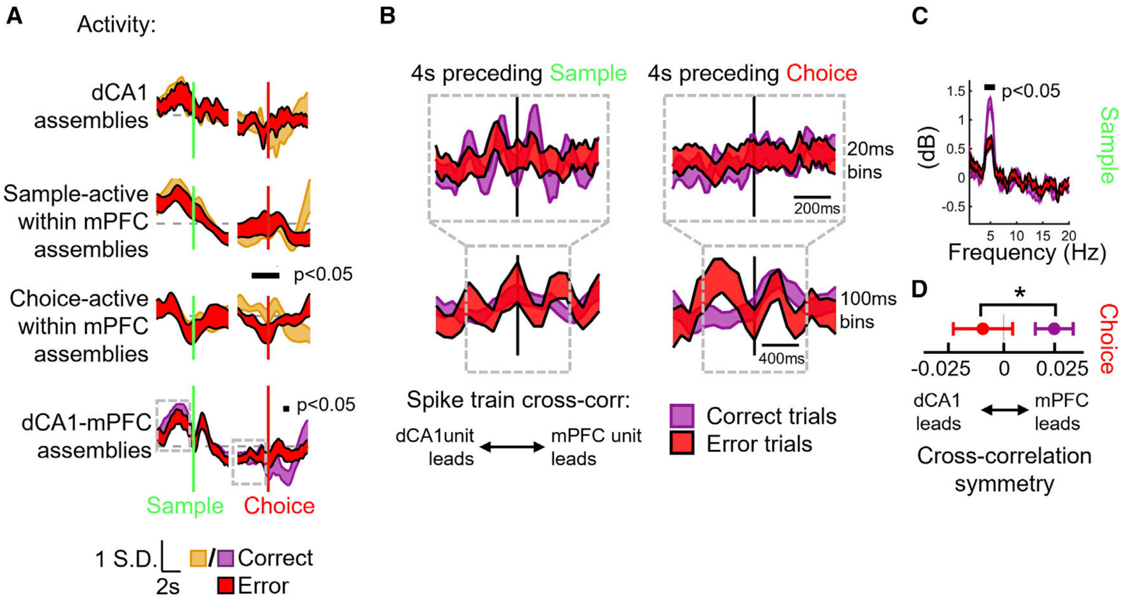

Figure 7. Incorrect choices are associated with intact sample encoding by dCA1 networks but reduced 4 Hz dCA1-mPFC assembly synchrony, leading to a collapse of intra-mPFC dynamics and impaired dCA1-mPFC synchronization on recall.

(A) Average z-scored activity of different classes of within- and inter-area cell assemblies on correct (gold/purple) and error (red) trials. Mean ± SEM across all pairs shown. Gray dashed lines indicate mean activity. Black bars mark times of significant changes on error trials (Bonferroni-corrected bootstrap $p < 0.05$, permutation test). Gray boxes indicate regions used for spike train cross-correlations in (C) and (D).

(B) Inter-area correlation between dCA1-mPFC cell pairs is presented from the same assembly on correct (purple) and error (red) trials (details as Figure 4D, average of 251 pairs from 10 sessions shown).

(C) Power spectrum of 20-ms-binned cross-correlations in 4 s preceding sample in (B) show frequencies with significant change in power between correct and error trials (black bars; Bonferroni-corrected bootstrap $p < 0.05$, permutation test).

(D) Balance of dCA1- to mPFC-driven correlation in 100-ms-binned spike times from the 4 s preceding choice was significantly reversed on error trials.

See also Figure S7.

structure of the operant DNMTS task, allowed us to deconstruct dCA1 and mPFC contributions during sample, delay, and choice stages: dCA1 populations provide earliest encoding of current task-relevant sensory information at sample, while mPFC populations preferentially maintain task-relevant information during delays[46] and are re-engaged during decisions. These differential dCA1 and mPFC coding patterns corroborate previous lesion studies in rodents,[89–91] population recordings in non-human primates,[73] and human imaging studies highlighting hippocampal activation during encoding of short-term memory.[56–59] However, we also resolved a physiologically distinct subset of dCA1 and mPFC neurons that proved critical during trial-specific loading of short-term memory (sample lever presses), coalescing into inter-regional assemblies coactive on sub-50-ms timescales.

Assembly activity during sampling presumably reflects hippocampal-prefrontal interactions during loading of short-term memory,[68] with the tendency of dCA1 encoding to precede mPFC encoding consistent with hippocampal-to-prefrontal anatomy[92–94] and functional connectivity (Figure 5D, see also[67]). However, the mPFC subpopulations recruited into dCA1-mPFC assemblies proved physiologically distinct from the mPFC subpopulations engaged in cue encoding during the subsequent DNMTS delay period. Delay-coding mPFC subpopulations may partner with mediodorsal thalamus, since optogenetic disruption of mPFC activity[95] or silencing mPFC input from mediodorsal thalamus during the delay phase of short-term memory tasks impairs maintenance of information.[24] Some recent evidence suggests that individual mPFC pyramidal neurons receive convergent input from both ventral CA1 and mediodorsal thalamus,[96] potentially enabling dynamic configuration of assemblies across task phases.

Our discovery of rhythmic modulation of dCA1-mPFC assembly participants is reminiscent of a 4-Hz rhythm previously reported to coordinate hippocampus, ventral tegmental area (VTA) and PFC during short-term memory processing in rats[97] and implicated in coordinating PFC-amygdala interactions during fear learning.[98] Although we do not dissect its source in the present study, we pinpoint its emergence during the sample phase, showing that it provides a distinct, second channel for CA1-mPFC communication beyond 8–10 Hz theta. In contrast to Fujisawa and Buzsaki,[97] who report both significantly tuned units and sustained 4-Hz LFP coherence during spatial navigation, we find only weak and transient 4–5 Hz LFP coherence associated with the lever press events. This fleeting population-level coherence in the DNMTS task is consistent with the sparse subset of 4–5 Hz modulated units that formed inter-regional cell assemblies.

4–5 Hz dCA1-mPFC assembly modulation was notably absent around DNMTS response lever presses (i.e., during choice-evoked use of short-term memory, Figure 4D), showing that hippocampal-prefrontal dynamics are reconfigured from sample to choice, potentially reflecting mPFC-led

control of memory retrieval.[54] Hippocampus projects directly to mPFC[92–94] and units in both structures exhibit 5 Hz intrinsic membrane resonance *in vitro*.[99,100] Lower frequency, 4–5 Hz oscillations may therefore act in concert with theta and gamma rhythms in subserving limbic-cortical communication, for instance, by tuning the resonant properties of selected neurons.[101]

The intrinsic time constants of mPFC neurons also influence the timescales over which they contribute to sustained information coding during short-term memory. Wasmuht et al.[102] show that the "temporal stability" (based on the decay time constant of an individual neuron's autocorrelation function) of primate prefrontal cortical neurons co-varies with their timing and duration of information coding during a short-term memory task.[35,45,74] However, in our rat mPFC data, the cross-temporal coding analyses in Figure 2 evidence sustained coding despite transient and dynamic activities of individual mPFC neurons. This delay coding was neither evident in dCA1, nor associated with the sustained or systematic activation of dCA1 or mPFC synchronous assemblies; it is most likely, therefore, to derive from sequential activation of mPFC units and/or assemblies.[46] Whatever its basis, sustained mPFC population coding during the DNMTS delay phase collapsed during errors, and on 16 s delay trials during sessions in which rats performed at overall chance levels.

Our results suggest that, although on error trials the dCA1 population code faithfully represents cue location (Figure 1H) and the assemblies that link dCA1 and mPFC are similarly active (Figure 7A) during both sample and choice epochs, transient rhythmic interactions that support transfer of this information for maintenance by the mPFC population code during the delay period are weaker (Figures 7B and 7C). This could lead to disorganized re-activation of the dCA1-mPFC cell assemblies when the rats are required to make a choice. Failures in the relay of sample information between dCA1 and mPFC by rhythmic coordination of assemblies during the sample encoding phase would therefore lead to aberrant network dynamics in mPFC during the delay (Figure 2C), preventing the formation of a stable population code for short-term memory and culminating in an incorrect choice. However, with the current dataset, we cannot dissect this causal sequence of individual signatures of errors.

Disrupted connectivity between the PFC and the hippocampus causes deficits in short-term memory[65,68,75,76,89,103,104] and is implicated in the pathophysiology of schizophrenia.[105,106] Our data show that the primary correlate of spontaneous errors was blunting of 4–5 Hz modulated dCA1-mPFC interactions during the sample phase of the task, while lever position coding in dCA1 remained intact. Rhythmic coordination across the limbic-cortical axis therefore remains a viable target for translational research into cognitive impairments in neuropsychiatry.

In conclusion, our data reveal why both mPFC and dCA1—as well as intact connectivity between them— have been ascribed crucial roles in spatial short-term memory: early encoding of trial-specific, sample information is strongest in dCA1 and integrated into mPFC processing by virtue of joint dCA1-mPFC assemblies, bound by a common 4–5 Hz rhythmic modulation. During the delays of up to 16 s used here, mPFC populations maintain sample information potentially through sequential activation tiling the delay; on error trials this coding peters out, despite accurate encoding in dCA1. Finally, dCA1 and mPFC concurrently encode choice information, led by mPFC, but only mPFC sustains this information beyond choice itself, potentially enabling the integration of trial outcome and the tuning of future responses. This temporally defined set of cognitive steps establishes a framework that can now be tested in combination with circuit tracing and/or imaging strategies relating assembly configurations to the connectivity of participating neurons and their neuromodulation.

STAR★METHODS

Detailed methods are provided in the online version of this paper and include the following:

- KEY RESOURCES TABLE
- RESOURCE AVAILABILITY
  - Lead contact
  - Materials availability
  - Data and code availability
- EXPERIMENTAL MODEL AND SUBJECT DETAILS
- METHOD DETAILS
  - Electrode implantation
  - Behavioral training
  - Single unit clustering
- QUANTIFICATION AND STATISTICAL ANALYSIS
  - Spike train analysis
  - Neural Decoding
  - FA model for cell assembly detection
  - Optimised synthetic cell assembly detection, benefits of noise coding
  - Coding distance

SUPPLEMENTAL INFORMATION

Supplemental information can be found online at https://doi.org/10.1016/j.cub.2023.02.029.

ACKNOWLEDGMENTS

The authors would like to thank the following funders for support: Medical Research Council (MRC), for an Industrial Collaborative Studentship with Eli Lilly & Co. Ltd. UK (M.T.K., M.D.T., and M.W.J.); The Wellcome Trust (Senior Research Fellowship in Basic Biomedical Science, 202810/Z/16/Z, MWJ); Ch. and H. Schaller Foundation and the Boehringer Ingelheim Foundation grant "Complex Systems" (E.R.); DFG (German Science Foundation) grants "Du 354/10-1" and "Du 354/8-2" (D.D.). Our thanks also to Debi Ford for expert histological assistance and to the University of Bristol Animal Services Unit staff for expert and caring rat husbandry.

AUTHOR CONTRIBUTIONS

M.T.K., M.W.J., E.S.J.R., and M.D.T. designed the experiments. M.T.K. collected the data. A.P.F.D., E.R., D.D., M.W.J., and M.T.K. developed methods for data analysis. A.P.F.D., E.R., and M.T.K. analyzed the data. A.P.F.D., E.R., D.D., M.W.J., and M.T.K. prepared figures for the manuscript. A.P.F.D., D.D., M.W.J., and M.T.K. wrote the manuscript. A.P.F.D., M.W.J., D.D., E.R., M.T.K, and M.D.T. edited the manuscript.

DECLARATION OF INTERESTS

The authors declare no competing interests.

CellPress
OPEN ACCESS

Received: September 20, 2022
Revised: January 5, 2023
Accepted: February 8, 2023
Published: March 9, 2023

REFERENCES

1. Baddeley, A. (2012). Working memory: theories, models, and controversies. Annu. Rev. Psychol. *63*, 1–29. https://doi.org/10.1146/annurev-psych-120710-100422.
2. Yu, J.Y., and Frank, L.M. (2015). Hippocampal-cortical interaction in decision making. Neurobiol. Learn. Mem. *117*, 34–41. https://doi.org/10.1016/j.nlm.2014.02.002.
3. Euston, D.R., Gruber, A.J., and McNaughton, B.L. (2012). The role of medial prefrontal cortex in memory and decision making. Neuron *76*, 1057–1070. https://doi.org/10.1016/j.neuron.2012.12.002.
4. Fuster, J.M. (1973). Unit activity in prefrontal cortex during delayed-response performance: neuronal correlates of transient memory. J. Neurophysiol. *36*, 61–78. https://doi.org/10.1152/jn.1973.36.1.61.
5. Funahashi, S., Bruce, C.J., and Goldman-Rakic, P.S. (1989). Mnemonic coding of visual space in the monkey's dorsolateral prefrontal cortex. J. Neurophysiol. *61*, 331–349. https://doi.org/10.1152/jn.1989.61.2.331.
6. Chafee, M.V., and Goldman-Rakic, P.S. (1998). Matching patterns of activity in primate prefrontal area 8a and parietal area 7ip neurons during a spatial working memory task. J. Neurophysiol. *79*, 2919–2940. https://doi.org/10.1152/jn.1998.79.6.2919.
7. Rainer, G., Asaad, W.F., and Miller, E.K. (1998). Selective representation of relevant information by neurons in the primate prefrontal cortex. Nature *393*, 577–579. https://doi.org/10.1038/31235.
8. Romo, R., Brody, C.D., Hernández, A., and Lemus, L. (1999). Neuronal correlates of parametric working memory in the prefrontal cortex. Nature *399*, 470–473. https://doi.org/10.1038/20939.
9. Hampson, R.E., and Deadwyler, S.A. (1996). Ensemble codes involving hippocampal neurons are at risk during delayed performance tests. Proc. Natl. Acad. Sci. USA *93*, 13487–13493. https://doi.org/10.1073/pnas.93.24.13487.
10. Hampson, R.E., and Deadwyler, S.A. (2003). Temporal firing characteristics and the strategic role of subicular neurons in short-term memory. Hippocampus *13*, 529–541. https://doi.org/10.1002/hipo.10119.
11. Hampson, R.E., Heyser, C.J., and Deadwyler, S.A. (1993). Hippocampal cell firing correlates of delayed-match-to-sample performance in the rat. Behav. Neurosci. *107*, 715–739. https://doi.org/10.1037/0735-7044.107.5.715.
12. Deadwyler, S.A., and Hampson, R.E. (1995). Ensemble activity and behavior: what's the code? Science *270*, 1316–1318. https://doi.org/10.1126/science.270.5240.1316.
13. Durstewitz, D., Vittoz, N.M., Floresco, S.B., and Seamans, J.K. (2010). Abrupt transitions between prefrontal neural ensemble states accompany behavioral transitions during rule learning. Neuron *66*, 438–448. https://doi.org/10.1016/j.neuron.2010.03.029.
14. Durstewitz, D., and Seamans, J.K. (2006). Beyond bistability: biophysics and temporal dynamics of working memory. Neuroscience *139*, 119–133. https://doi.org/10.1016/j.neuroscience.2005.06.094.
15. Falco, E. De, An, L., Sun, N., Roebuck, A.J., Greba, Q., Lapish, C.C., and Howland, J.G. (2019). The rat medial prefrontal cortex exhibits flexible neural activity states during the performance of an odor span task. eNeuro *6*. ENEURO.0424-18.2019.
16. Funahashi, S. (2015). Functions of delay-period activity in the prefrontal cortex and mnemonic scotomas revisited. Front. Syst. Neurosci. *9*, 2. https://doi.org/10.3389/fnsys.2015.00002.
17. Constantinidis, C., Funahashi, S., Lee, D., Murray, J.D., Qi, X.L., Wang, M., and Arnsten, A.F.T. (2018). Persistent spiking activity underlies working memory. J. Neurosci. *38*, 7020–7028. https://doi.org/10.1523/JNEUROSCI.2486-17.2018.
18. Wang, X.J. (2001). Synaptic reverberation underlying mnemonic persistent activity. Trends Neurosci. *24*, 455–463. https://doi.org/10.1016/s0166-2236(00)01868-3.
19. Zylberberg, J., and Strowbridge, B.W. (2017). Mechanisms of persistent activity in cortical circuits: possible neural substrates for working memory. Annu. Rev. Neurosci. *40*, 603–627. https://doi.org/10.1146/annurev-neuro-070815-014006.
20. Bouchacourt, F., and Buschman, T.J. (2019). A flexible model of working memory. Neuron *103*, 147–160.e8. https://doi.org/10.1016/j.neuron.2019.04.020.
21. Hart, E., and Huk, A.C. (2020). Recurrent circuit dynamics underlie persistent activity in the macaque frontoparietal network. eLife *9*, 1–22. https://doi.org/10.7554/eLife.52460.
22. Parnaudeau, S., O'Neill, P.K., Bolkan, S.S., Ward, R.D., Abbas, A.I., Roth, B.L., Balsam, P.D., Gordon, J.A., and Kellendonk, C. (2013). Inhibition of mediodorsal thalamus disrupts thalamofrontal connectivity and cognition. Neuron *77*, 1151–1162. https://doi.org/10.1016/j.neuron.2013.01.038.
23. Wimmer, R.D., Schmitt, L.I., Davidson, T.J., Nakajima, M., Deisseroth, K., and Halassa, M.M. (2015). Thalamic control of sensory selection in divided attention. Nature *526*, 705–709. https://doi.org/10.1038/nature15398.
24. Bolkan, S.S., Stujenske, J.M., Parnaudeau, S., Spellman, T.J., Rauffenbart, C., Abbas, A.I., Harris, A.Z., Gordon, J.A., and Kellendonk, C. (2017). Thalamic projections sustain prefrontal activity during working memory maintenance. Nat. Neurosci. *20*, 987–996. https://doi.org/10.1038/nn.4568.
25. Rikhye, R.V., Gilra, A., and Halassa, M.M. (2018). Thalamic regulation of switching between cortical representations enables cognitive flexibility. Nat. Neurosci. *21*, 1753–1763. https://doi.org/10.1038/s41593-018-0269-z.
26. Hauer, B.E., Pagliardini, S., and Dickson, C.T. (2019). The reuniens nucleus of the Thalamus Has an essential role in coordinating slow-wave activity between neocortex and hippocampus. eNeuro *6*, 0365–19.2019. eNeuro. https://doi.org/10.1523/ENEURO.0365-19.2019.
27. Schmitt, L.I., Wimmer, R.D., Nakajima, M., Happ, M., Mofakham, S., and Halassa, M.M. (2017). Thalamic amplification of cortical connectivity sustains attentional control. Nature *545*, 219–223. https://doi.org/10.1038/nature22073.
28. Raghavachari, S., Kahana, M.J., Rizzuto, D.S., Caplan, J.B., Kirschen, M.P., Bourgeois, B., Madsen, J.R., and Lisman, J.E. (2001). Gating of human theta oscillations by a working memory task. J. Neurosci. *21*, 3175–3183. https://doi.org/10.1523/JNEUROSCI.21-09-03175.2001.
29. Roberts, B.M., Hsieh, L.T., and Ranganath, C. (2013). Oscillatory activity during maintenance of spatial and temporal information in working memory. Neuropsychologia *51*, 349–357. https://doi.org/10.1016/j.neuropsychologia.2012.10.009.
30. Motes, M.A., and Rypma, B. (2010). Working memory component processes: isolating BOLD signal changes. NeuroImage *49*, 1933–1941. https://doi.org/10.1016/j.neuroimage.2009.08.054.
31. Riggall, A.C., and Postle, B.R. (2012). The relationship between working memory storage and elevated activity as measured with functional magnetic resonance imaging. J. Neurosci. *32*, 12990–12998. https://doi.org/10.1523/JNEUROSCI.1892-12.2012.
32. Rottschy, C., Langner, R., Dogan, I., Reetz, K., Laird, A.R., Schulz, J.B., Fox, P.T., and Eickhoff, S.B. (2012). Modelling neural correlates of working memory: a coordinate-based meta-analysis. NeuroImage *60*, 830–846. https://doi.org/10.1016/j.neuroimage.2011.11.050.
33. Sreenivasan, K.K., Curtis, C.E., and D'esposito, M. (2014). Revisiting the role of persistent neural activity during working memory. Trends Cogn. Sci. *18*, 82–89. https://doi.org/10.1016/j.tics.2013.12.001.
34. Lundqvist, M., Herman, P., and Miller, E.K. (2018). Working memory: delay activity, yes! Persistent Activity? Maybe Not. J. Neurosci. *38*, 7013–7019. https://doi.org/10.1523/JNEUROSCI.2485-17.2018.

35. Cavanagh, S.E., Towers, J.P., Wallis, J.D., Hunt, L.T., and Kennerley, S.W. (2018). Reconciling persistent and dynamic hypotheses of working memory coding in prefrontal cortex. Nat. Commun. *9*, 3498. https://doi.org/10.1038/s41467-018-05873-3.

36. Harvey, C.D., Coen, P., and Tank, D.W. (2012). Choice-specific sequences in parietal cortex during a virtual-navigation decision task. Nature *484*, 62–68. https://doi.org/10.1038/nature10918.

37. Rajan, K., Harvey, C.D., and Tank, D.W. (2016). Recurrent network models of sequence generation and memory. Neuron *90*, 128–142. https://doi.org/10.1016/j.neuron.2016.02.009.

38. Park, J.C., Bae, J.W., Kim, J., and Jung, M.W. (2019). Dynamically changing neuronal activity supporting working memory for predictable and unpredictable durations. Sci. Rep. *9*, 15512. https://doi.org/10.1038/s41598-019-52017-8.

39. Murray, J.D., Bernacchia, A., Roy, N.A., Constantinidis, C., Romo, R., and Wang, X.J. (2017). Stable population coding for working memory coexists with heterogeneous neural dynamics in prefrontal cortex. Proc. Natl. Acad. Sci. USA *114*, 394–399. https://doi.org/10.1073/pnas.1619449114.

40. Loewenstein, Y., and Sompolinsky, H. (2003). Temporal integration by calcium dynamics in a model neuron. Nat. Neurosci. *6*, 961–967. https://doi.org/10.1038/nn1109.

41. Hasselmo, M.E., and Stern, C.E. (2006). Mechanisms underlying working memory for novel information. Trends Cogn. Sci. *10*, 487–493. https://doi.org/10.1016/j.tics.2006.09.005.

42. Mongillo, G., Barak, O., and Tsodyks, M. (2008). SynaptiC theory of working memory. Science *319*, 1543–1546. https://doi.org/10.1126/science.1150769.

43. Vogels, T.P., and Abbott, L.F. (2005). Signal propagation and logic gating in networks of integrate-and-fire neurons. J. Neurosci. *25*, 10786–10795. https://doi.org/10.1523/JNEUROSCI.3508-05.2005.

44. Druckmann, S., and Chklovskii, D.B. (2012). Neuronal circuits underlying persistent representations despite time varying activity. Curr. Biol. *22*, 2095–2103. https://doi.org/10.1016/j.cub.2012.08.058.

45. Stokes, M.G. (2015). "Activity-silent" working memory in prefrontal cortex: A dynamic coding framework. Trends Cogn. Sci. *19*, 394–405. https://doi.org/10.1016/j.tics.2015.05.004.

46. Baeg, E.H., Kim, Y.B., Huh, K., Mook-Jung, I., Kim, H.T., and Jung, M.W. (2003). Dynamics of population code for working memory in the prefrontal cortex. Neuron *40*, 177–188. https://doi.org/10.1016/S0896-6273(03)00597-X.

47. Balaguer-Ballester, E., Lapish, C.C., Seamans, J.K., and Durstewitz, D. (2011). Attracting dynamics of frontal cortex ensembles during memory-guided decision-making. PLoS Comput. Biol. *7*, e1002057. https://doi.org/10.1371/journal.pcbi.1002057.

48. Spaak, E., Watanabe, K., Funahashi, S., and Stokes, M.G. (2017). Stable and dynamic coding for working memory in primate prefrontal cortex. J. Neurosci. *37*, 6503–6516. https://doi.org/10.1523/JNEUROSCI.3364-16.2017.

49. Leavitt, M.L., Pieper, F., Sachs, A.J., and Martinez-Trujillo, J.C. (2017). Correlated variability modifies working memory fidelity in primate prefrontal neuronal ensembles. Proc. Natl. Acad. Sci. USA *114*, E2494–E2503. https://doi.org/10.1073/pnas.1619949114.

50. Rigotti, M., Barak, O., Warden, M.R., Wang, X.-J.J., Daw, N.D., Miller, E.K., and Fusi, S. (2013). The importance of mixed selectivity in complex cognitive tasks. Nature *497*, 585–590. https://doi.org/10.1038/nature12160.

51. Parthasarathy, A., Herikstad, R., Bong, J.H., Medina, F.S., Libedinsky, C., and Yen, S.-C.C. (2017). Mixed selectivity morphs population codes in prefrontal cortex. Nat. Neurosci. *20*, 1770–1779. https://doi.org/10.1038/s41593-017-0003-2.

52. Balaguer-Ballester, E., Nogueira, R., Abolafia, J.M., Moreno-Bote, R., and Sanchez-Vives, M.V. (2020). Representation of foreseeable choice outcomes in orbitofrontal cortex triplet-wise interactions. PLoS Comput. Biol. *16*, e1007862. https://doi.org/10.1371/journal.pcbi.1007862.

53. Watanabe, T., and Niki, H. (1985). Hippocampal unit activity and delayed response in the monkey. Brain Res. *325*, 241–254. https://doi.org/10.1016/0006-8993(85)90320-8.

54. Navawongse, R., and Eichenbaum, H. (2013). Distinct pathways for rule-based retrieval and spatial mapping of memory representations in hippocampal neurons. J. Neurosci. *33*, 1002–1013. https://doi.org/10.1523/JNEUROSCI.3891-12.2013.

55. Goonawardena, A.V., Robinson, L., Riedel, G., and Hampson, R.E. (2010). Recruitment of hippocampal neurons to encode behavioral events in the rat: alterations in cognitive demand and cannabinoid exposure. Hippocampus *20*, 1083–1094. https://doi.org/10.1002/hipo.20706.

56. Gluth, S., Sommer, T., Rieskamp, J., and Büchel, C. (2015). Effective connectivity between hippocampus and ventromedial prefrontal cortex controls preferential choices from memory. Neuron *86*, 1078–1090. https://doi.org/10.1016/j.neuron.2015.04.023.

57. Roberts, B.M., Libby, L.A., Inhoff, M.C., and Ranganath, C. (2018). Brain activity related to working memory for temporal order and object information. Behav. Brain Res. *354*, 55–63. https://doi.org/10.1016/j.bbr.2017.05.068.

58. Günseli, E., and Aly, M. (2020). Preparation for upcoming attentional states in the hippocampus and medial prefrontal cortex. eLife *9*, 1–33. https://doi.org/10.7554/eLife.53191.

59. Newmark, R.E., Schon, K., Ross, R.S., and Stern, C.E. (2013). Contributions of the hippocampal subfields and entorhinal cortex to disambiguation during working memory. Hippocampus *23*, 467–475. https://doi.org/10.1002/hipo.22106.

60. Kamiński, J., and Rutishauser, U. (2020). Between persistently active and activity-silent frameworks: novel vistas on the cellular basis of working memory. Ann. N. Y. Acad. Sci. *1464*, 64–75. https://doi.org/10.1111/nyas.14213.

61. Jin, J., and Maren, S. (2015). Prefrontal-hippocampal interactions in memory and emotion. Front. Syst. Neurosci. *9*, 170. https://doi.org/10.3389/fnsys.2015.00170.

62. Sigurdsson, T., and Duvarci, S. (2015). Hippocampal-Prefrontal Interactions in Cognition, Behavior and Psychiatric Disease. Front. Syst. Neurosci. *9*, 190. https://doi.org/10.3389/fnsys.2015.00190.

63. Jones, M.W., and Wilson, M.A. (2005). Theta rhythms coordinate hippocampal-prefrontal interactions in a spatial memory task. PLoS Biol. *3*, e402. https://doi.org/10.1371/journal.pbio.0030402.

64. Benchenane, K., Peyrache, A., Khamassi, M., Tierney, P.L., Gioanni, Y., Battaglia, F.P., and Wiener, S.I. (2010). Coherent theta oscillations and reorganization of spike timing in the hippocampal- prefrontal network upon learning. Neuron *66*, 921–936. https://doi.org/10.1016/j.neuron.2010.05.013.

65. O'Neill, P.K., Gordon, J.A., and Sigurdsson, T. (2013). Theta oscillations in the medial prefrontal cortex are modulated by spatial working memory and synchronize with the hippocampus through its ventral subregion. J. Neurosci. *33*, 14211–14224. https://doi.org/10.1523/JNEUROSCI.2378-13.2013.

66. Myroshnychenko, M., Seamans, J.K., Phillips, A.G., and Lapish, C.C. (2017). Temporal dynamics of hippocampal and medial prefrontal cortex interactions during the delay period of a working memory-guided foraging task. Cereb. Cortex *27*, 5331–5342. https://doi.org/10.1093/cercor/bhx184.

67. Place, R., Farovik, A., Brockmann, M., and Eichenbaum, H. (2016). Bidirectional prefrontal-hippocampal interactions support context-guided memory. Nat. Neurosci. *19*, 992–994. https://doi.org/10.1038/nn.4327.

68. Spellman, T., Rigotti, M., Ahmari, S.E., Fusi, S., Gogos, J.A., and Gordon, J.A. (2015). Hippocampal-prefrontal input supports spatial encoding in working memory. Nature *522*, 309–314. https://doi.org/10.1038/nature14445.

69. Sloan, H.L., Good, M., and Dunnett, S.B. (2006). Double dissociation between hippocampal and prefrontal lesions on an operant delayed matching task and a water maze reference memory task. Behav. Brain Res. *171*, 116–126. https://doi.org/10.1016/j.bbr.2006.03.030.

70. Fujisawa, S., Amarasingham, A., Harrison, M.T., and Buzsáki, G. (2008). Behavior-dependent short-term assembly dynamics in the medial prefrontal cortex. Nat. Neurosci. *11*, 823–833. https://doi.org/10.1038/nn.2134.

71. Cowen, S.L., and McNaughton, B.L. (2007). Selective delay activity in the medial prefrontal cortex of the rat: contribution of sensorimotor information and contingency. J. Neurophysiol. *98*, 303–316. https://doi.org/10.1152/jn.00150.2007.

72. Brincat, S.L., and Miller, E.K. (2015). Frequency-specific hippocampal-prefrontal interactions during associative learning. Nat. Neurosci. *18*, 576–581. https://doi.org/10.1038/nn.3954.

73. Liu, Y., Brincat, S.L., Miller, E.K., and Hasselmo, M.E. (2020). A geometric characterization of population coding in the prefrontal cortex and hippocampus during a paired-associate learning task. J. Cogn. Neurosci. *32*, 1455–1465.

74. Meyers, E.M., Freedman, D.J., Kreiman, G., Miller, E.K., and Poggio, T. (2008). Dynamic population coding of category information in inferior temporal and prefrontal cortex. J. Neurophysiol. *100*, 1407–1419. https://doi.org/10.1152/jn.90248.2008.

75. Maharjan, D.M., Dai, Y.Y., Glantz, E.H., and Jadhav, S.P. (2018). Disruption of dorsal hippocampal – prefrontal interactions using chemogenetic inactivation impairs spatial learning. Neurobiol. Learn. Mem. *155*, 351–360. https://doi.org/10.1016/j.nlm.2018.08.023.

76. Schmidt, B., Duin, A.A., and Redish, A.D. (2019). Disrupting the medial prefrontal cortex alters hippocampal sequences during deliberative decision making. J. Neurophysiol. *121*, 1981–2000. https://doi.org/10.1152/jn.00793.2018.

77. Krzanowski, W.J. (2000). Principles of Multivariate Analysis: A User's Perspective (Oxford University Press).

78. Durstewitz, D. (2017). Advanced Data Analysis in Neuroscience (Springer).

79. Yu, B.M., Cunningham, J.P., Santhanam, G., Ryu, S.I., Shenoy, K.V., and Sahani, M. (2009). Gaussian-process factor analysis for low-dimensional single-trial analysis of neural population activity. J. Neurophysiol. *102*, 614–635. https://doi.org/10.1152/jn.90941.2008.

80. Chae, S., Sohn, J.W., and Kim, S.P. (2022). Investigation of neural substrates of erroneous behavior in a delayed-response task. eNeuro *9*, 1–16. https://doi.org/10.1523/ENEURO.0490-21.2022.

81. Lopes-dos-Santos, V., Ribeiro, S., and Tort, A.B.L. (2013). Detecting cell assemblies in large neuronal populations. J. Neurosci. Methods *220*, 149–166. https://doi.org/10.1016/J.JNEUMETH.2013.04.010.

82. Abbott, L.F., and Dayan, P. (1999). The effect of correlated variability on the accuracy of a population code. Neural Comput. *11*, 91–101. https://doi.org/10.1162/089976699300016827.

83. Backen, T., Treue, S., and Martinez-Trujillo, J.C. (2018). Encoding of spatial attention by primate prefrontal cortex neuronal ensembles. eNeuro *5*, 0372–16.2017. https://doi.org/10.1523/ENEURO.0372-16.2017.

84. Tremblay, S., Pieper, F., Sachs, A., and Martinez-Trujillo, J. (2015). Attentional filtering of visual information by neuronal ensembles in the primate lateral prefrontal cortex. Neuron *85*, 202–215. https://doi.org/10.1016/j.neuron.2014.11.021.

85. Xia, M., Liu, T., Bai, W., Zheng, X., and Tian, X. (2019). Information transmission in HPC-PFC network for spatial working memory in rat. Behav. Brain Res. *356*, 170–178. https://doi.org/10.1016/j.bbr.2018.08.024.

86. Liu, T., Bai, W., Xia, M., and Tian, X. (2018). Directional hippocampal-prefrontal interactions during working memory. Behav. Brain Res. *338*, 1–8. https://doi.org/10.1016/j.bbr.2017.10.003.

87. Deadwyler, S.A., Bunn, T., and Hampson, R.E. (1996). Hippocampal ensemble activity during spatial delayed-nonmatch-to-sample performance in rats. J. Neurosci. *16*, 354–372. https://doi.org/10.1523/JNEUROSCI.16-01-00354.1996.

88. Hyman, J.M., Zilli, E.A., Paley, A.M., and Hasselmo, M.E. (2010). Working memory performance correlates with prefrontal-hippocampal theta interactions but not with prefrontal neuron firing rates. Front. Integr. Neurosci. *4*, 2. https://doi.org/10.3389/neuro.07.002.2010.

89. Izaki, Y., Takita, M., and Akema, T. (2008). Specific role of the posterior dorsal hippocampus-prefrontal cortex in short-term working memory. Eur. J. Neurosci. *27*, 3029–3034. https://doi.org/10.1111/j.1460-9568.2008.06284.x.

90. Yoon, T., Okada, J., Jung, M.W., and Kim, J.J. (2008). Prefrontal cortex and hippocampus subserve different components of working memory in rats. Learn. Mem. *15*, 97–105. https://doi.org/10.1101/lm.850808.

91. Churchwell, J.C., Morris, A.M., Musso, N.D., and Kesner, R.P. (2010). Prefrontal and hippocampal contributions to encoding and retrieval of spatial memory. Neurobiol. Learn. Mem. *93*, 415–421. https://doi.org/10.1016/j.nlm.2009.12.008.

92. Laroche, S., Davis, S., and Jay, T.M. (2000). Plasticity at hippocampal to prefrontal cortex synapses: dual roles in working memory and consolidation. Hippocampus *10*, 438–446. https://doi.org/10.1002/1098-1063(2000)10:4<438::AID-HIPO10>3.0.CO;2-3.

93. Thierry, A.M., Gioanni, Y., Dégénétais, E., and Glowinski, J. (2000). Hippocampo-prefrontal cortex pathway: anatomical and electrophysiological characteristics. Hippocampus *10*, 411–419. https://doi.org/10.1002/1098-1063(2000)10:4<411::AID-HIPO7>3.0.CO;2-A.

94. Hoover, W.B., and Vertes, R.P. (2007). Anatomical analysis of afferent projections to the medial prefrontal cortex in the rat. Brain Struct. Funct. *212*, 149–179. https://doi.org/10.1007/s00429-007-0150-4.

95. Gilmartin, M.R., Miyawaki, H., Helmstetter, F.J., and Diba, K. (2013). Prefrontal activity links nonoverlapping events in memory. J. Neurosci. *33*, 10910–10914. https://doi.org/10.1523/JNEUROSCI.0144-13.2013.

96. Canetta, S., Teboul, E., Holt, E., Bolkan, S.S., Padilla-Coreano, N., Gordon, J.A., Harrison, N.L., and Kellendonk, C. (2020). Differential synaptic dynamics and circuit connectivity of hippocampal and thalamic inputs to the prefrontal cortex. Cereb. Cortex Commun. *1*, tgaa084. https://doi.org/10.1093/texcom/tgaa084.

97. Fujisawa, S., and Buzsáki, G. (2011). A 4 Hz oscillation adaptively synchronizes prefrontal, VTA, and hippocampal activities. Neuron *72*, 153–165. https://doi.org/10.1016/j.neuron.2011.08.018.

98. Karalis, N., Dejean, C., Chaudun, F., Khoder, S., Rozeske, R.R., Wurtz, H., Bagur, S., Benchenane, K., Sirota, A., Courtin, J., and Herry, C. (2016). 4-Hz oscillations synchronize prefrontal-amygdala circuits during fear behavior. Nat. Neurosci. *19*, 605–612. https://doi.org/10.1038/nn.4251.

99. Dembrow, N.C., Chitwood, R.A., and Johnston, D. (2010). Projection-specific neuromodulation of medial prefrontal cortex neurons. J. Neurosci. *30*, 16922–16937. https://doi.org/10.1523/JNEUROSCI.3644-10.2010.

100. Zemankovics, R., Szabolcs, K., Paulsen, O., Freund, F., and Norbert, H. (2011). Differences in subthreshold resonance of hippocampal pyramidal cells and interneurons: the role of h-current and passive membrane characteristics. J. Physiol. *588*, 2109–2132. https://doi.org/10.1113/jphysiol.2009.185975.

101. Hutcheon, B., and Yarom, Y. (2000). Resonance, oscillation and the intrinsic frequency preferences of neurons. Trends Neurosci. *23*, 216–222.

102. Wasmuht, D.F., Spaak, E., Buschman, T.J., Miller, E.K., and Stokes, M.G. (2018). Intrinsic neuronal dynamics predict distinct functional roles during working memory. Nat. Commun. *9*, 3499. https://doi.org/10.1038/s41467-018-05961-4.

103. Floresco, S.B., Seamans, J.K., and Phillips, A.G. (1997). Selective roles for hippocampal, prefrontal cortical, and ventral striatal circuits in radial-arm maze tasks with or without a delay. J. Neurosci. *17*, 1880–1890. https://doi.org/10.1523/JNEUROSCI.17-05-01880.1997.

104. Kucewicz, M.T., Tricklebank, M.D., Bogacz, R., and Jones, M.W. (2011). Dysfunctional prefrontal cortical network activity and interactions following cannabinoid receptor activation. J. Neurosci. *31*, 15560–15568. https://doi.org/10.1523/JNEUROSCI.2970-11.2011.

105. Meyer-Lindenberg, A.S., Olsen, R.K., Kohn, P.D., Brown, T., Egan, M.F., Weinberger, D.R., and Berman, K.F. (2005). Regionally specific disturbance of dorsolateral prefrontal-hippocampal functional connectivity in schizophrenia. Arch. Gen. Psychiatry *62*, 379–386. https://doi.org/10.1001/archpsyc.62.4.379.

106. Sigurdsson, T., Stark, K.L., Karayiorgou, M., Gogos, J.A., and Gordon, J.A. (2010). Impaired hippocampal–prefrontal synchrony in a genetic mouse model of schizophrenia. Nature *464*, 763–767. https://doi.org/10.1038/nature08855.

107. Paxinos, G., and Watson, C. (2007). The Rat Brain in Stereotaxic Coordinates, Sixth Edition: Hard Cover Edition (Academic Press).

108. Taylor, C.C. (1989). Bootstrap choice of the smoothing parameter in kernel density estimation. Biometrika *76*, 705–712.

109. Shimazaki, H., and Shinomoto, S. (2010). Kernel bandwidth optimization in spike rate estimation. J. Comput. Neurosci. *29*, 171–182.

110. Hastie, T., Tibshirani, R., and Friedman, J. (2011). The Elements of Statistical Learning: Data Mining, Inference, and Prediction (Springer).

111. Stokes, M.G., Kusunoki, M., Sigala, N., Nili, H., Gaffan, D., and Duncan, J. (2013). Dynamic coding for cognitive control in prefrontal cortex. Neuron *78*, 364–375. https://doi.org/10.1016/j.neuron.2013.01.039.

112. Cunningham, J.P., and Yu, B.M. (2014). Dimensionality reduction for large-scale neural recordings. Nat. Neurosci. *17*, 1500–1509. https://doi.org/10.1038/nn.3776.

CellPress
OPEN ACCESS

STAR★METHODS

KEY RESOURCES TABLE

REAGENT or RESOURCE	SOURCE	IDENTIFIER
Experimental models: Organisms/strains		
Rat: 300-400g male Long Evans	Harlan, UK	HsdBlu:LE
Software and algorithms		
https://github.com/apfdomanski/Domanski_CurrentBiology_2023	Our work for this project	https://doi.org/10.6084/m9.figshare.21944759.v1

RESOURCE AVAILABILITY

Lead contact
Further information and requests for resources and reagents should be directed to and will be fulfilled by the lead contact, Michal T. Kucewicz (michal.kucewicz@pg.edu.pl).

Materials availability
This study did not generate new unique reagents.

Data and code availability

- All experimental data reported in this paper will be made available online with open access: https://data.bris.ac.uk/data/ and shared upon request from the lead contact
- All original code has been deposited at github and is publicly available as of the date of publication from: https://github.com/apfdomanski/Domanski_CurrentBiology_2023. DOIs are listed in the key resources table.
- Any additional information required to reanalyze the data reported in this paper is available from the lead contact upon request.

EXPERIMENTAL MODEL AND SUBJECT DETAILS

All procedures were conducted in accordance with the UK Animals Scientific Procedures Act (1986) and with the approval of the University of Bristol Ethics Committee. This study used a total of 8 adult (300–400g) male Long–Evans rats (Harlan UK).

METHOD DETAILS

Electrode implantation
Seven adult male rats were implanted with 16 extracellular tetrode recording electrodes: 8 over right medial prefrontal cortex (+3.2 mm, +0.6 mm from bregma) and 8 over the right dorsal hippocampus (−4.0 mm, +2.5 mm from bregma) under sodium pento-barbital recovery anaesthesia. Data are presented from 6 of the 8 rats; one failed to learn the task, so was not implanted and one implant failed shortly after surgery. During 7–12 days following surgery the independently moveable tetrodes were lowered into pre-limbic cortex (~2–3 mm ventral) and the principal cell layer of the dCA1,[107] guided by the characteristic burst mode of single-unit firing and the presence of large-amplitude sharp-wave ripple events in the local field potential. Extracellular action potentials (sampled at 32 kHz and filtered between 0.6–6 kHz) together with local field potentials (sampled at 2 kHz and filtered between 0.1–475 Hz) were recorded differentially (Digital Lynx, Neuralynx) using local references, which were targeted to superficial prefrontal cortex and the white matter overlying the hippocampus. Two screws placed in the skull overlying the cerebellum were used as ground connections. Final tetrode tip positions were verified histologically in 4/6 rats (Figure S1C) by identifying sites of electrolytic lesions in 50um stained sections of formaldehyde-perfused brain; lesioning failed in 2/6 rats, but coordinates and results were consistent across animals.

Behavioral training
Subjects were food-restricted to no less than 85% of their free-feeding weight and trained in a DNMTS operant task (Figure 1). We used an operant chamber (Med-Associates, Vermont, USA), which consisted of two retractable levers facing a food pellet dispenser on the opposite wall, with a cue light above each component and a tone generator placed above the pellet dispenser. Every trial began with a sample phase initiated by presentation of one lever on either the right or left side of the operant chamber wall, cued with a light above the presented lever. Following sample press, the lever was automatically retracted and rats turned and waited in front of a food pellet receptacle at the opposite wall until the end of a 4, 8 or 16 s delay (varying randomly from trial to trial to

discourage mediating behavior), signalled by a 500ms tone. The choice phase was initiated by nose-poking inside the receptacle after the tone; nose-poking triggered insertion of both levers into the chamber on the opposite wall. Correct choice was rewarded according to a non-match rule.

Side and top walls of the chamber were transparent to enable view of distal spatial cues in the recording room. Metal components of the chamber were grounded to the amplifier to electrically shield the recordings, which were carried to the data acquisition system via tethers suspended through a hole in the centre of the box ceiling. The task was programmed and operated in K-Limbic software (D. Fuller, Conclusive Marketing Ltd.) on a separate computer. Subjects were initially conditioned to press a lever to obtain pellet reward before being trained in DNMTS task with pseudo-random delays (random combination of equal number of target left and right lever trails at each delay arranged into shuffled blocks of 10 trials) of 4,8, 16s. Error and missed trials were followed by all cue lights off for an extra 10s of inter-trial interval. There were 150 trials in each session (50 x 3 delays). Sessions with less than 67% of trials completed were excluded from further analysis.

Single unit clustering

Single units were isolated off-line using automated clustering software (KlustaKwik 1.7; K. Harris), followed by verification and manual refinement in Mclust 3.5 (A.D. Redish); unit inclusion criteria were set to isolation distance >10.0 and L-ratio <0.35, with <2% of spikes within 2ms inter-spike interval. Putative pyramidal cells were classified based on the spike width, waveform and mean firing rate. A total of 156 (min. 115, max. 194) putative principal cells in dCA1 (mean of 34 units per subject) and 168 (min. 152, max. 201) putative pyramidal cells in mPFC (mean of 33 units per subject) were isolated in each recording session.

QUANTIFICATION AND STATISTICAL ANALYSIS

Where appropriate following normality testing (Kolmogorov-Smirnov test, p>0.05), parametric statistical comparisons were performed. Unless otherwise specified, results are quoted as Mean ± Standard Error of the Mean (SEM). To equalize statistical power on multivariate statistical analyses, all comparisons across delay lengths and different recording sessions were calculated on repeated jack-knife draws of random subsets of matched numbers of trials. Similarly, non-parametric bootstrapping was performed through calculating statistics on distributions of shuffled data (e.g. for decoding analyses described below, by randomly permuting trial labels 1000 times). Results were considered significant if the observed value exceeded the 95th percentile of the bootstrap distribution. Two bootstrap resampled distributions were considered significantly different if their <5% and >95% tails did not overlap. Where two time series were compared (e.g. Figures 1G and 1H), bootstrapped p–values were adjusted using Bonferroni correction for number of time bins.

Spike train analysis

Only units with an average firing rate of at least 0.5Hz were included in all subsequent analyses. All decoding analyses, to be described further below, were performed on kernel density estimates of the instantaneous spiking rate. Separate kernel density estimates (KDE) for each unit ***I*** were obtained by convolving spike trains with Gaussian functions ('kernels'), where the optimal kernel width σ^2 was determined through unbiased cross-validation.[108] For Gaussian kernels, closed-form expressions for the unbiased cross-validation error (CVE) can be obtained, and numerical iteration of the CVE procedure is not necessary.[108] Loosely, one may think of the unbiased cross-validation procedure as leaving out each spike in turn, and evaluating the likelihood of its actual position from the spike density estimate obtained based on all other spikes in the series. Thus, the optimal bandwidth estimated will depend on predictable temporal structure in the spike trains, not just their rate (see also [109]. KDEs provide a statistically more robust (less variable) estimate of the true underlying spike density, compared to e.g. histograms or binarized spike series, but decoding results did not crucially depend on this pre-processing step.

Single units were considered significantly sensitive to behavioural events if their normalized (z-scored) firing rate deflection in a 2 second window after the event exceeded ±3x the standard deviation of the baseline firing rate (500ms window preceding the event).

Neural Decoding

For single unit decoding (e.g. Figure 1D), for each time bin m and unit i single unit rates v_{im} were collected into two sets according to whether $m \in C1$ or $m \in C2$, the two sets of time bins associated with one (C1) or the other (C2) cue stimulus. The common t-statistic (as also employed in Student's two-sample t-test) is a measure of discrimination among these two sets, as it divides the difference in means by the pooled standard deviation (c.f. [13]). For the average number of trials collected here (~54), values of approximately $t > 1.67$ would indicate significant discrimination at the $p<0.05$ level.

Leave-one-out cross-validation analysis (e.g. Figures 1G and 1H) was performed for the multivariate linear discriminant classifiers used for decoding (e.g. [110]), with regularized covariance matrix as specified below. This used, for each time bin t the two sets of population vectors associated with the two stimulus classes (see above), with one population vector (and thus trial) left out from the fitting. Prediction performance was evaluated on the left-out trial, and this was repeated for each trial in turn, yielding the cross-validation error (CVE) as the relative number of incorrectly classified (out-of-sample) prediction trials. For testing differences in CVE between mPFC and dCA1 populations, for each data set the number of mPFC and dCA1 units (variables) used for decoding was exactly equalized to rule out any potential confounds due to population size. This was done by fixing the number K of units used to the smaller of the two populations, mPFC or dCA1, and then randomly drawing K units with replacement from the larger of the two populations 10 times

and averaging the obtained CVE values. Differences in relative proportions of correct cue predictions, CP = 1-CVE $\in$ [0, 1], between mPFC and dCA1 were statistically tested by averaging CP across all 12 data sets and using the beta distribution. Specifically, at each time point *t* the smaller of the two values CP_{mPFC} and CP_{dCA1} was used for the reference distribution, and it was checked whether the larger of the two significantly ($p<0.05$) escaped this reference distribution given the average number of trials recorded.

To compare decoding performance on correct and error trials (e.g. Figure 1H), classifiers trained on correct trials were additionally challenged to predict the cue identity of error trials in a similar manner as above.

To evaluate the stability of the population code for cue location during the delay period we used cross-temporal decoding methods inspired by.[51,111] Briefly, we performed leave-one-out cross-validated decoding of cue location from multi- single unit firing rates as described above using separate training and testing sets offset by sequential 50ms increments. The performance of the decoder at each combination of [train,test] time points is thus the percentage of test trials in which the decoder could correctly identify the cue location when trained using trials at a given time point.

To equalize size of training sets across combinations of delay lengths and recording sessions, cross-temporal decoding analysis was performed on random draws of eight trials from each of left and right cue conditions, repeated 500 times. Thus the total cross-validation size for each decoder was 5000 random resamples per [train,test] time combination. Mean performance across runs is reported in the colormaps shown in (e.g.) Figure 2B. Significant decoding at each [train,test] point was calculated against distributions ($p<0.05$) from 1000 bootstrap draws created by shuffling labels. For visualization, a 250ms Gaussian smoothing kernel was applied after significance testing across both training and testing dimensions.

To further corroborate the classification results, we also used a parametric test statistic (Figure S6B): Vectors $\mathbf{v}_m=(\mathbf{v}_{1l}.\mathbf{v}_{pm})^T$ of all unit activities were collected into two sets corresponding to stimulus conditions as above, and contrasted by Hotelling's T^2 statistic, a multivariate generalization of the univariate two-sample t-statistic which relates differences in cue specific mean vectors to the pooled covariance matrix of the data.[77] Hotelling's T^2, scaled by the appropriate degrees of freedom, is approximately F-distributed which can be used to construct parametric confidence bands.

In the present case, the number of recorded units often reaches or even exceeds the number of trials, causing singularity and over-fitting issues with the covariance matrix. One standard statistical remedy is regularization, where the covariance matrix Σ is moved toward the identity, $\Sigma_{reg} = \Sigma + \lambda I$, with regularization parameter λ(set to 0.05 here, without any attempt to optimise this parameter[110]).

FA model for cell assembly detection

Extraction of cell assemblies was based on Factor Analysis.[78] This model-based statistical tool is designed to extract *correlations* between variables. It assumes that observation vectors $\mathbf{v}_{i,t}$ are given by a (linear) mixing of uncorrelated latent variables (factors) $\mathbf{z}_{i,t}$, plus common mean μ_i and measurement noise $\varepsilon_{i,t}$,

$$\mathbf{V}_{i,t} = \mu_i + \Gamma \mathbf{z}_{i,t} + \varepsilon,\ \varepsilon_{i,t} \sim N(\mathbf{0},\mathbf{\Psi}),\ \mathbf{z}_{i,t} \sim N(0,\mathbf{I}),\ \mathbf{\Psi} = \mathrm{diag}[\sigma_1^2,\ldots,\sigma_N^2].$$

Parameters are commonly estimated through maximum-likelihood. Unlike principal component analysis (PCA) which detects variance-maximizing directions, FA attempts to capture all the *correlations* among the observed variables through the mixing of uncorrelated factors (e.g.[77]). It is thus more appropriate for assembly detection than PCA, as has been demonstrated before.[79,112]

Inputs to FA were the kernel density estimated instantaneous firing rate vectors $\mathbf{c}_m=(c_{im})$ which collected spike rates c_{im} for each ulit *i* at time *m* binned at 50ms in columns, excised from time periods from cue presentation -5s to choice lever press +5s, combined from all trials.

Each of the 12 recorded data sets was treated separately, with simultaneously recorded mPFC only, dCA1 only, or concatenated mPFC and dCA1 units submitted for assembly analysis.

The likelihood-ratio statistic for FA models of increasing complexity (i.e. increasing number of factors) in conjunction with confidence bands obtained from trial-shuffled data can be used to determine the number of putative assemblies (i.e. significant factors) present (Figure 3B). While, in principle, likelihood-ratio based parametric F-scores could be used to determine whether adding another factor to the model still significantly improves the fit, here we relied on H_0 distributions generated from trial permutation bootstraps to account for the time series (and thus potentially dependent) nature of the data. Specifically, if $\mathbf{c}_i^{(k)} = (c_{i1}^{(k)} \ldots c_{iM}^{(k)})$ denotes the set of firing rates for Init *i* on trial *k*, for each unit separately the assignments of these sets to trials *k* were randomly shuffled. Thus, all autocorrelations and the firing rate structure across a trial were preserved for each unit *i* in the bootstrap data, while cross-dependencies between units were destroyed. These bootstrapped data sets (total of 500) were used both to determine the number of significant factors, i.e. those for which the LLR ranged within the 1% upper confidence limit of the bootstrap data, as well as significant factor loadings (the correlations of the units with the factors): Only units for which a *factor loading* exceeded 1% of the bootstrap range were assigned to the respective assembly. For each factor, the *factor score* (the value z_{lm} on factor *l* in time bin *m*) quantifies the degree to which the respective assembly is activated. Local cell assemblies forming subsets of joint area assemblies were assumed as part of the larger assembly.

Assembly detection by FA was confirmed using another method based on Independent Component Analysis.[81] Assembly units were determined using IC weight threshold of 2.5 S.D. for every spike train in the analysis. As done for the FA based analysis, neuronal assemblies, defined as groups of three or more single units that consistently co-activated within a 50ms time window, were thus detected in dCA1, mPFC, and across dCA1 and mPFC. Despite this quite different methodological approach, sets of assemblies detected by ICA were highly similar to those detected by FA as quantified through the measure of overlap $O=|A\cap B|/|A\cup B| \in [0,1]$ between pairs of sets as defined further above. For each assembly set detected by ICA, the corresponding assembly set with highest

similarity to it as detected by FA was first determined, and the average across O from all these ICA x FA pairs then calculated for each data set. Overall, across all data sets, there was an 84% average agreement between FA and ICA assemblies (Figure S3A). We considered whether to penalize by assembly size, or neuron pool sizes: As well as using bootstrapped assembly size detection, in an alternative detection validation step (data not shown) we also tried Bayesian Information Criterion (BIC) as a metric to detect significant assembly formation amongst neurons, penalizing for number of degrees of freedom in the latent factor model (i.e. no. neurons involved in the assembly). Quantitatively similar results were observed.

Optimised synthetic cell assembly detection, benefits of noise coding

To investigate the optimality of cue encoding by FA-detected cell assemblies, we compared the multivariate leave-one-out CVE of assembly member units against that of sub-sampled groups of units drawn from available single units. This procedure was performed independently for each of the 12 recording sessions, using both mPFC and dCA1 as well firing rate matrices concatenated across areas. Curves in Figure 4E thus represent the mean performance of these draws across sessions. Single units were first ranked in descending order based on their individual peak CVE in the time span ±5s surrounding the sample lever press and the 5s before the choice lever press. Groups of size 2∼20 single units were drawn from this ranked matrix of firing rates for the best, 2nd best, 3rd best, …, 10th best draw for each synthetic draw pool size, or until available unit pools were exceeded. CVE decoding was performed using jack-knife trial subsamples as performed above, and the mean peak decoding performance (% correct decoding of withheld trial cue label) was calculated in the same time window as for individual single units. Decoding performance of multivariate decoder for each FA-detected cell assembly member units was thus compared to that of size-matched groups of single units. FA-detected assembly performance was finally described as that of the closest-performing ranked synthetic assembly.

To estimate the contributions of within-trial ‘noise’ correlations in firing rates to cue location coding, decoding performance was compared as a function of assembly size against the performance of decoding on the same drawn pool of units in which the cue labels were maintained but where successive trial labels had been shuffled (average of 50 permutations), either within or between areas. An alternative construction method of searching amongst all potential pairs, triplets, quads etc., to find optimal assemblies produced qualitatively similar results (data not shown).

Coding distance

To compare the similarity of cue information carried by firing rates of single units, or activities of cell assemblies (factor scores), we calculated the mean pairwise distance between their t-score profiles after matched trial count univariate decoding performed as outlined above. Mean Euclidean coding distance was then calculated between pairs of units/assemblies following scaling by pooled variance and removing mean offset. Alternative vector-based distance metrics (Cosine distance, Correlation) produced quantitatively similar results.

Current Biology, Volume *33*

Supplemental Information

Distinct hippocampal-prefrontal neural assemblies coordinate memory encoding, maintenance, and recall

Aleksander P.F. Domanski, Michal T. Kucewicz, Eleonora Russo, Mark D. Tricklebank, Emma S.J. Robinson, Daniel Durstewitz, and Matt W. Jones

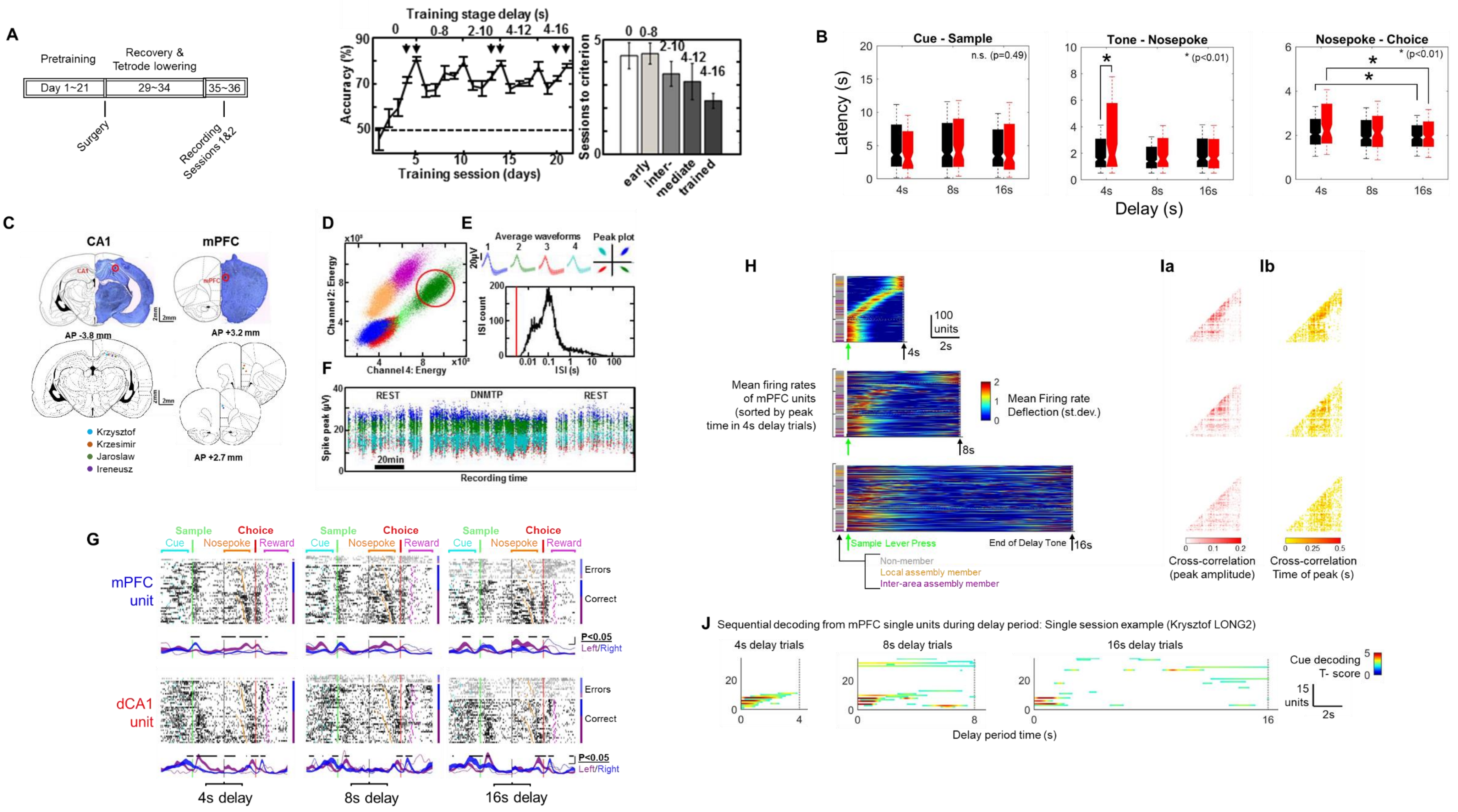
A
Pretraining
Recovery & Tetrode lowering
Day 1~21
29~34
35~36
Surgery
Recording Sessions 1&2
Training stage delay (s)
0
0-8
2-10
4-12
4-16
Accuracy (%)
Training session (days)
Sessions to criterion
early
inter-mediate
trained
B
Cue - Sample
n.s. (p=0.49)
Tone - Nosepoke
* (p<0.01)
Nosepoke - Choice
* (p<0.01)
Latency (s)
Delay (s)
4s
8s
16s
C
CA1
mPFC
AP -3.8 mm
AP +3.2 mm
AP +2.7 mm
2mm
Krzysztof
Krzesimir
Jaroslaw
Ireneusz
D
Channel 2: Energy
Channel 4: Energy
E
Average waveforms
Peak plot
20μV
ISI count
ISI (s)
F
Spike peak (μV)
REST
DNMTP
REST
20min
Recording time
G
mPFC unit
dCA1 unit
Sample
Choice
Cue
Nosepoke
Reward
Errors
Correct
P<0.05
Left/Right
4s delay
8s delay
16s delay
H
Mean firing rates of mPFC units (sorted by peak time in 4s delay trials)
100 units
2s
Mean Firing rate Deflection (st.dev.)
Sample Lever Press
End of Delay Tone
Non-member
Local assembly member
Inter-area assembly member
Ia
Ib
Cross-correlation (peak amplitude)
Cross-correlation Time of peak (s)
J
Sequential decoding from mPFC single units during delay period: Single session example (Krysztof LONG2)
4s delay trials
8s delay trials
16s delay trials
Delay period time (s)
Cue decoding T- score
15 units
2s

Figure S1: Details of training, recordings and single unit physiology during DNMTS task (relating to Figure 1).

A Left: Experimental timeline, Center: Behavioral performance of rats in DNMTS training is expressed as choice accuracy across subsequent sessions of the training and as average number of sessions required to reach criterion performance at different stages of training: early (Right). Arrows mark the two sessions used in the analysis of each stage (early, intermediate, trained). Data from the "trained" days is analysed in this study.
B Cue-Sample, Delay-Nosepoke, Nosepoke-Choice latencies averaged across animals and recording sessions for correct (black) and error (red) trials. Asterisks at upper right corner indicate significant difference between conditions (Kruskal-Wallis ANOVA: $\chi^{!}$(5,1751)=3.69/16.29/16.53, $p>0.05$/$p<0.01$/$p<0.01$, respectively). Bars/asterisks indicate significant differences between specific combinations of delay and outcome conditions (Tukey-Kramer post-hoc test, $p<0.05$).
C Coronal sections show example locations of tetrode recording sites (red circles mark the site of electrolytic lesions) in the prelimbic cortex (mPFC) and in the pyramidal cell layer of the dCA1 subfield in dorsal hippocampus (dCA1), matched to a corresponding rat brain atlas schematic (from Paxinos 2008). The lower panels summarize lesion sites across all six rats.
D Extracellular action potential spikes recorded across an entire session were clustered into separate single units (colored dots), plotted here as waveform energy recorded on two channels of one mPFC tetrode. The properties of the cluster in red circle are presented in E and F.
E Mean waveforms recorded on color-coded channels of the tetrode (top left) show consistent relative peak amplitudes (top right). Distribution of inter-spike intervals (ISI) below show no spikes detected in the <2ms refractory period.
F Spike peak amplitudes of the same unit recorded on the color-coded four tetrode channels remain stable across the recording session.
G Multi-trial firing raster from one example mPFC (top) and dCA1 (bottom) single units. Spike rasters with continuous firing rates aligned to the Sample and Choice lever presses +/-5s, with a variable portion of the delay period excised depending on delay length. Trials are sorted by correct and error outcomes (black and grey ticks) as well as left and right trial type, and finally by nose-poke latency. Solid areas indicate mean +/-SEM firing rates on correct trials, dotted lines show mean firing rate on error trials. Black bars above epochs show significant separation of Left/Right trial responses, from the trial-averaged firing rates (t-score, in 50ms non-overlapping increments, Bonferroni-corrected).
H Mean firing rates of mPFC units (preferred cue direction, correct trials, all units across sessions combined) during the delay period sorted by time of peak firing on 4s delay trials, sort order maintained for 8,16s delay trials. Colored stripes on Left indicate assembly membership class (See Figures 3,4).
I Firing rate correlation matrices (Ia: peak correlation and Ib: time-lag at peak) for data shown in H. Heat-map shows mean correlation across trials, note strong non-zero lagged correlations.
J Sequential contributions of individual mPFC single units to maintaining population-level encoding of cue location during maintenance delay. Single recording session shown. Units are shown sorted by center-of-mass of significant decoding (bootstrapped Bonferroni $p<0.05$) on 4s delay trials. Sort times maintained across longer delay lengths. Colourmap as for Figure 1D.

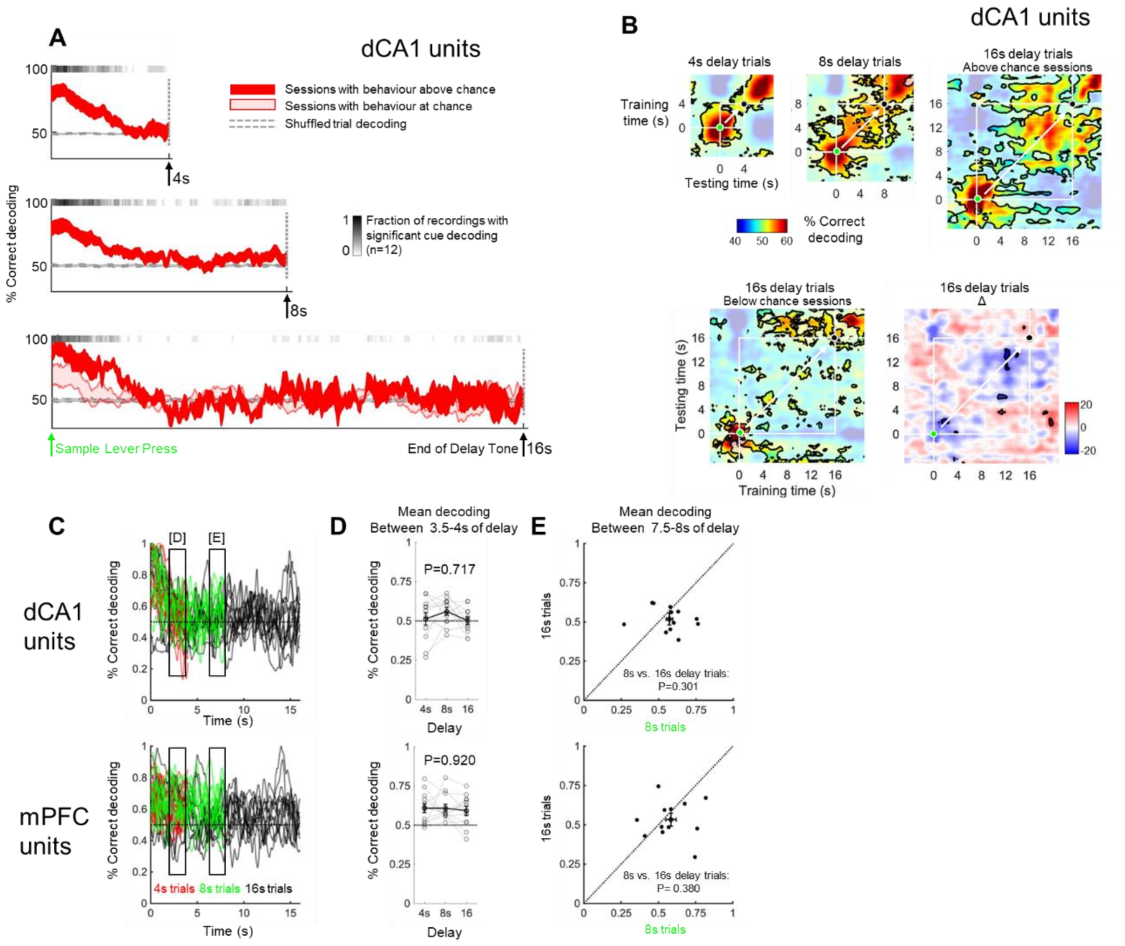

Figure S2: Transient cue encoding in dCA1 population lacks a stable code, further details of delay coding in dCA1 and mPFC (relating to Figure 2).

A-B: Encoding of cue information during the delay. Legends as for Figure 2 but decoding from populations of dCA1 single units.
C: Delay-dependent performance of dCA1 and mPFC populations for each session, regardless of whether the rat was performing above or at chance for that delay length (each line represents on session). Black boxes indicate the 0.5s windows used to calculate the average decoding performance per dCA1/mPFC population around 4s and 8s during the delay (shown in D and E, respectively).
D: No trial-dependent differences in average decoding between 3.5-4s during the delay were detected in either brain area. Linked symbols are recording sessions, linked by delay length. Error bars indicate mean±SEM (Friedman's test: dCA1: c_2(2,22)=0.67, p=0.717; mPFC: c_2(2,22)=0.17, p=0.920).
E: No trial-dependent differences in average decoding between 7.5-8s during the delay were detected in either brain area. Linked symbols are recording sessions, linked by delay length. Error bars indicate mean±SEM. Paired Wilcoxon dCA1: T= 25, z = -1.10, p=0.301, mPFC: T = 32, z = -0.550,P=0.380.

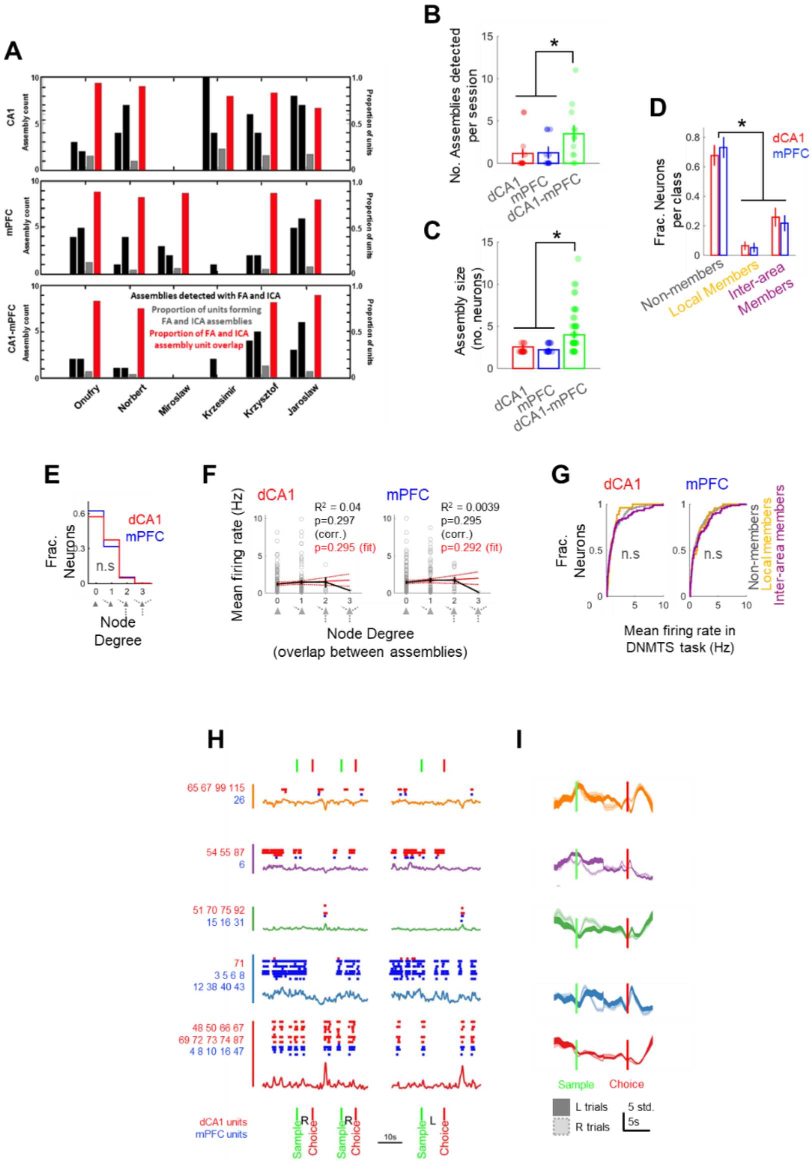

Figure S3: Further details of detected local and inter-regional dCA1-mPFC cell assemblies (relating to Figure 3).

A Validation of assemblies detected with the FA against PCA-ICA based methods. Total number of assemblies detected (black bars – the first for FA, second for ICA), proportion of units that participated in assemblies detected by both FA and ICA relative to all units recorded (grey), and proportion of unit overlap between matched FA and ICA assembly pairs (red) are summarized for the two sessions of each rat. A measure of overlap between a pair of assembly sets A and B was formally defined as O=|AÇB|/|AČB|î [0,1], i.e. the cardinality of the intersection divided by the cardinality of the union. On average there was 84% overlap between units detected by the FA and ICA methods, with similar numbers for total counts and unit proportions involved.
B Detection rates for local and inter-regional classes of cell assemblies. Symbols are individual sessions (1.2±0.5/1.3±0.7 dCA1/mPFC vs. 3.5±0.9 joint dCA1-mPFC Kruskal-Wallis test: C2(2,33)=6.69 p=0.035 with Bonferroni-correct post-hoc test).
C Size of detected cell assemblies, for dCA1/mPFC/inter-area classes, ANOVA F(2,16)=6,2,N=71 p=0.01).
D Breakdown of units by assembly membership class A significant minority of units participated in cell assemblies (ANOVA for member vs, non-member, F(5,66)=32.3, p<0.01, local membership: 6±3%/5±3%; dCA1/mPFC vs inter-area membership 26±3%/22±5% of dCA1/mPFC neurons). Assembly membership was similarly sparse in both dCA1 and mPFC area: Fraction of members vs. non-members dCA1 vs mPFC: C2(1)=0.33, p=0.94.
E The majority of neurons forming cell assemblies did so with little overlap between membership: 73%/67% of dCA1/mPFC units were detected as members of only a single assembly, with no differences in membership orthogonality observed between the two areas

(Figure 3F, KS test for node degree of mPFC vs. dCA1 neurons, D=0.045, p=0.87, N=295;317, dC1;mPFC).

F Mean firing rates of units calculated across the duration of the task were not affected by membership degree (Figure 3G, red curves: Linear fit vs. no relationship, dCA1: F(270)=1.09, p=0.30, adjusted R^2=3x10^{-4}; F(287)=1.11, p=0.29, adjusted R^2=4x10^{-4}). Not shown: Firign rate did not depend on global assembly membership (Mann-Whitney U-test for mean firing rate of members vs non-members: p=0.6,p=0.63 for dCA1,mPFC neurons, respectively, N=295,317 neurons).

G Classifying neurons as non-members, local members or inter-area cell assembly members did not significantly partition mean firing rate distributions (Figure 3H, Kruskal Wallis test: dCA1: C^2(2,292)=0.29, p=087; mPFC: C^2(2,314)=0.33, p=0.86). Similar results were obtained from surrogate measure of optimal firing rate smoothing kernel (data not shown).

H Activities of co-occurring inter dCA1-mPFC cell assemblies from example recording shown in Figure 3C-D. Legend as for Figure 3D: units from each area are shown concatenated.

I Normalized trial-averaged activity of assemblies in H during sample and choice lever events, for Left and Right cue trials. Mean±SEM activity across trials shown.

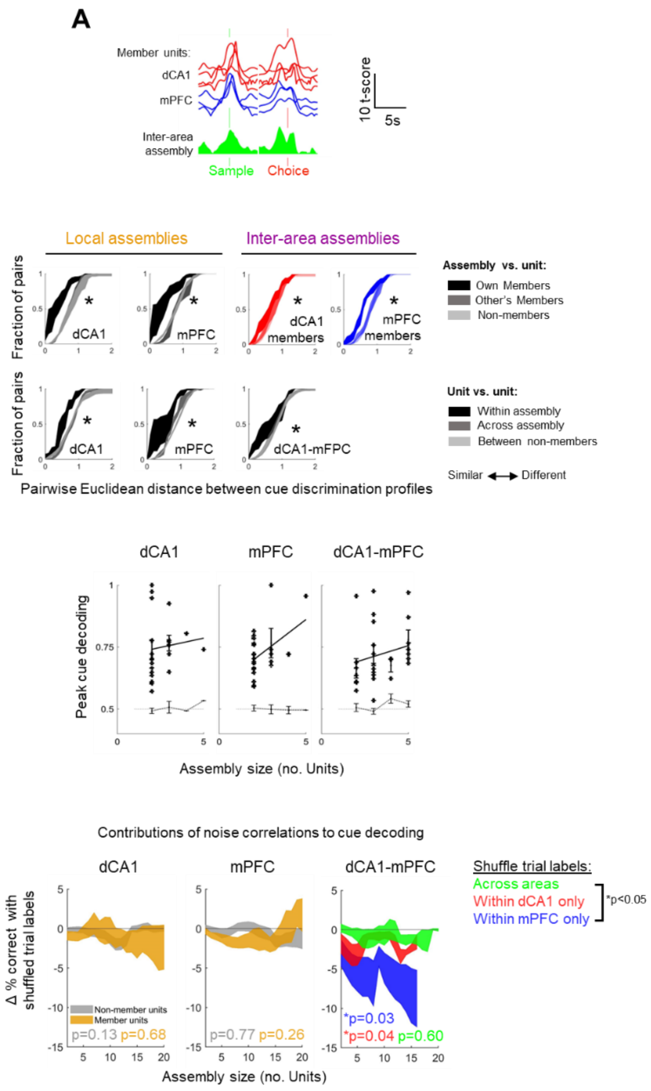

Figure S4: Cell assemblies link units carrying similar information (relating to Figure 4).

A Cue location decoding profiles of an example dCA1-mPFC assembly and its constituent member units aligned to Sample and Choice lever presses.
B Distributions of pairwise Euclidean distances between cue-decoding profiles of pairs of units/assemblies, sorted by assembly membership comparison type. In all cases, distances are closer (temporal evolution is more similar) for within- than between-assembly comparisons (Mean±SEM across sessions shown, asterisks indicates Kruskal-Wallis test, $p<0.05$).
C Expanded x range of Figure 4F, showing relationship between cell assembly size and peak cue-decoding.
D Contributions of within-trial ('noise') firing rate correlations to performance of optimally aggregated cell assemblies shown in Figure 4F. Mean±SEM peak performance change of best performing synthetic cell assemblies after shuffling trial labels, keeping cue location labels intact (Negative: reduced decoding performance after removing within-trial correlations). Colored p-values indicate one-sample t-test results: Within-area: dCA1 non-members: $t(7)=-1.21$, $p=0.13$; members: $t(8)=0.42$, $p=0.68$; mPFC non-members: $t(11)=-0.29$, $p=0.77$; members: $t(11)=-1.63$, $p=0.26$. Inter-area: within-dCA1: $t(7) =-2.75$, $p=0.04$; within-mPFC: $t(7) = -2.63$, $p = 0.04$; cross-dCA1-mPFC: $t(9) = -0.54$, $p=0.60$. Right: contributions of within-trial firing rate correlations of inter-area assembly performance were significantly different within and across areas (ANOVA: $F(2,23)=4.02$, $p=0.032$); cue decoding after ablation within mPFC pool caused significantly greater impairment than across dCA1-mPFC correlations (Asterisk:Tukey-Kramer post-hoc test, $p<0.05$).

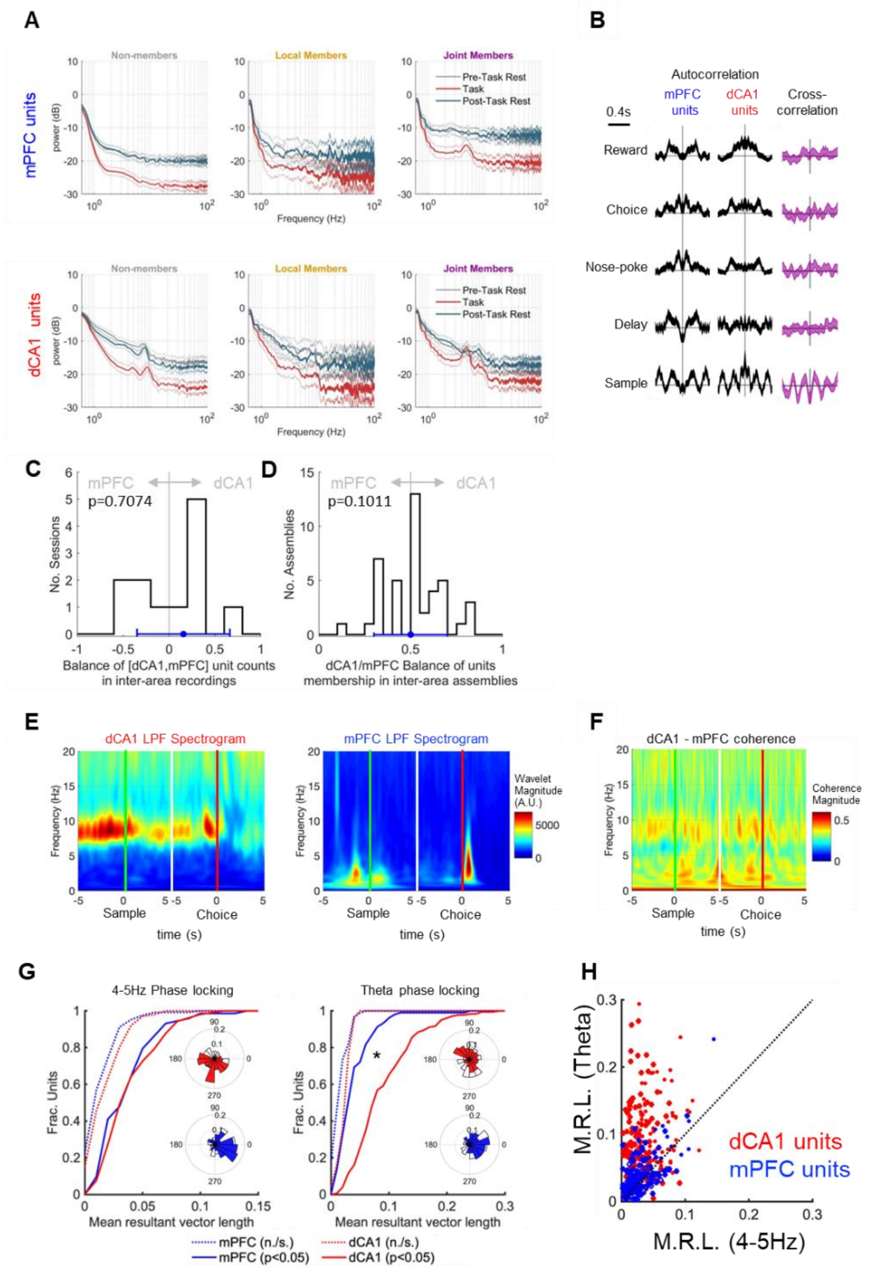

Figure S5: Physiological details of dCA1-mPFC cell assemblies (relating to Figure 5).

A Power spectral densities of spike time autocorrelations for units of each area sorted by assembly membership category. Curves show mean±SEM spectra, Spikes are drawn from either the task period (red), or 1h pre- and post-task rest periods (grey, teal). For both dCA1 and mPFC units, the 4-5Hz oscillation was specific to the task period and, in mPFC, to crossregional assembly members.
B Left: Z-scored spike time autocorrelation (black) and cross-correlation (purple) functions of dCA1 and mPFC units restricted to task events. Lines show mean±SEM.
C Units from both regions are equally represented in our recordings: Histogram across sessions of ratios between single unit counts in multi-area recordings $[(\#units_{"\#\$\%} - \#units_{\&\%'()})/(\#units_{"\#\$\%} + \#units_{\&\%'()})]$. Ratio distribution was not significantly different from a normal distribution (Kolmogorov-Smirnoff test). Blue symbols indicate median ± inter-quartile range.
D Inter-area cell assemblies are equally contributed by dCA1 and mPFC single units. Ratio distribution was not significantly different from a normal distribution with mean=0.5. (Kolmogorov-Smirnoff test). Blue symbols indicate median ± inter-quartile range.
E Mean wavelet spectrograms of dCA1 (left) and mPFC (right) LFP, aligned to the sample and choice lever press events in the task. Average of one tetrode per session for each area.
F As for E, but showing wavelet coherogram between dCA1 an mPFC LFP signals.
G Single unit phase locking to 4-5Hz (left) and 8-12Hz (“theta”, right) LFP oscillations during

the DNMTS task. Cumulative histograms show distributions of phase locking strengths (mean resultant length of phase vectors) for units with insignificant (dotted) and significant (solid) phase locking (Rayleigh's test $p<0.05$). Insets show distributions of peak phase preferences for units.

H Relationship between strengths of phase locking to 4-5Hz and theta LFP oscillations for single units which showed significant phase preference for both rhythms.

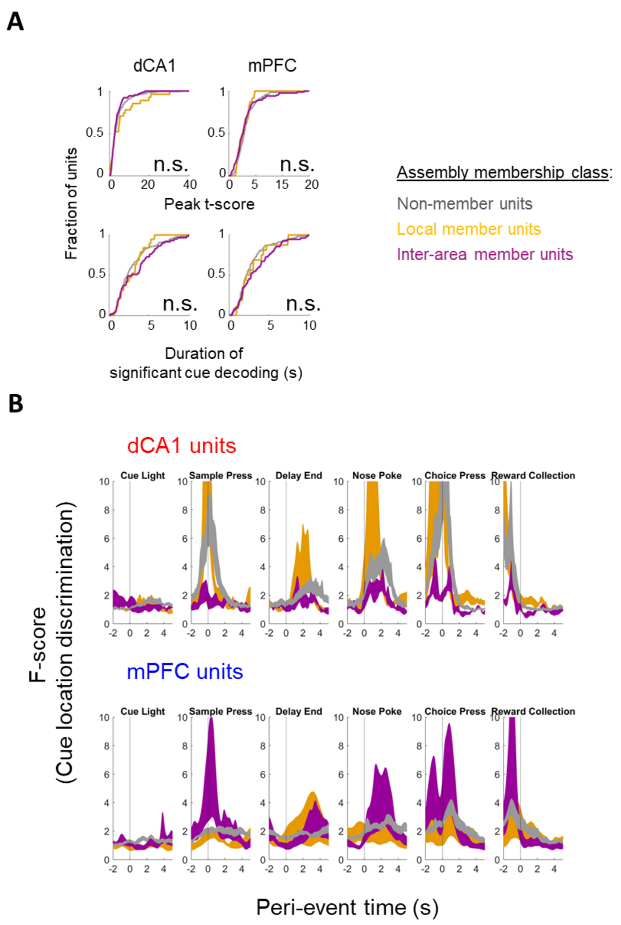

Figure S6: Assembly membership influences population but not single cell properties (relating to Figure 6).

A Classifying dCA1 (left) and mPFC (right) single units as non-members, local members or inter-area cell assembly members did not significantly affect cumulative distributions of peak strength (top) or duration (bottom) of significant cue encoding in the DNMTS task (Kruskal-Wallis ANOVA, Bonferroni-corrected $p>0.05$).
B Evolution of cue discrimination during the DNMTS task by populations of dCA1 and mPFC units is determined by cell assembly membership participation. Time-aligned multivariate cue discrimination (regularised F-scores) of populations of each type of units for each event in the DNMTS task. Shaded regions indicate mean±SEM F-scores for units of each membership classification, from the 12 recording sessions (see methods for details).

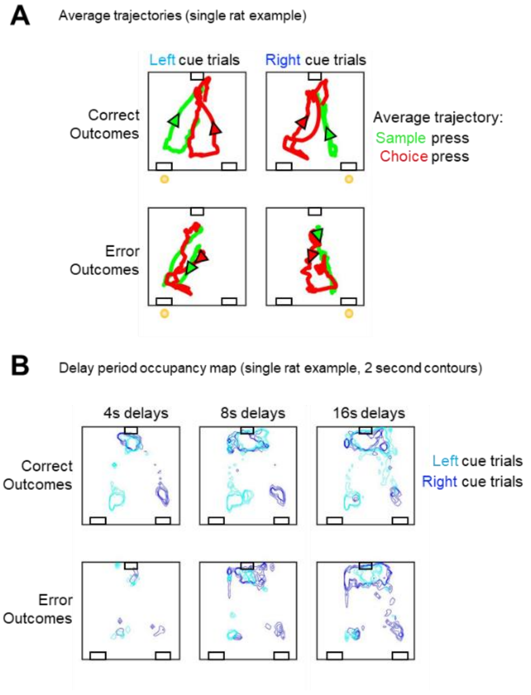

Figure S7: Details of behavior during errors (relating to Figure 7).

A Average spatial trajectories surrounding (±4s) sample and choice lever press events for one example rat on correct and error trials
B Cumulative dwell time (spatial occupancy) of example rat during delays in the DNMTS task on correct and error trials.
